# Supplementary material for: Protocol for a multicentre prospective exploratory mixed-methods study investigating the modifiable psychosocial variables influencing access to and outcomes after kidney transplantation in children and young people in the UK
Source: BMJ Open. 2024 May 28;14(5):e078150. doi: 10.1136/bmjopen-2023-078150 (PMC11138291; doi:10.1136/bmjopen-2023-078150)
Supplement: Supplementary data [file bmjopen-2023-078150supp001.pdf]

Supplementary Material 1

An investigation of the modifiable psychosocial variables influencing access to and outcomes after kidney transplantation in children

Interview Topic Guide for Researcher – with parent / guardian

Research Goals of the Interview:

1. Explore how the participant feels their life is now (with End Stage Kidney Disease [ESKD])
2. Explore what good quality life looks like to the participant – what matters most?
3. Explore what the participant believes is delaying or enabling their child to receive a kidney transplant

[1] Introduction, Overview:

- *Welcome, introduction of the research project and the researcher*
- *Establish a 'stop sign' for research participant to use if needing to pause the interview*

[2] Discussion of…:

Note for Interviewer: Subjects covered in this topic guide are to be explored openly – not leading. Appropriate prompts will be used – e.g. can you tell me more about that? How did that make you feel?

1. What is day-to-day living with ESKD like? (exploring different aspects of daily life – family life, school/college, employment, any treatment, limitations, participation in activities etc)
2. Which aspects of daily-living really matter to a family living with ESKD?
3. What are their experiences and beliefs around preparing for/receiving a kidney transplant?

[3] Conclusion:

1. *Summarise what has been discussed, mention the positive details, thank the young person / parent / carer*
2. "How did you find talking about living with chronic kidney disease?"
3. "Is there anything important to YOU that we have not talked about?"
4. Handle any concerns they have, signpost to any local services that could support their concerns.

Kim JS, *et al. BMJ Open* 2024; 14:e078150. doi: 10.1136/bmjopen-2023-078150

An investigation of the modifiable psychosocial variables influencing access to and outcomes after kidney transplantation in children

Interview Topic Guide for Researcher – with the Young Person

Research Goals of the Interview:

4. Explore how the participant feels their life is now (with End Stage Kidney Disease [ESKD])
5. Explore what good quality life looks like to the participant – what matters most?
6. Explore what the participant believes is delaying or enabling the young person to receive a kidney transplant

[1] Introduction, Overview:

- *Welcome, introduction of the research project and the researcher*
- *Establish a 'stop sign' for research participant to use if needing to pause the interview*

[2] Discussion of…:

Note for Interviewer: Subjects covered in this topic guide are to be explored openly – not leading. Appropriate prompts will be used – e.g. can you tell me more about that? How did that make you feel?

4. What is day-to-day living with ESKD like? (exploring different aspects of daily life – family life, school/college, any treatment, limitations, participation in activities etc)
5. Which aspects of daily-living really matter to a family living with ESKD?
6. What are their experiences and beliefs around preparing for/receiving a kidney transplant?

[3] Conclusion:

5. *Summarise what has been discussed, mention the positive details, thank the young person / parent / carer*
6. "How did you find talking about living with chronic kidney disease?"
7. "Is there anything important to YOU that we have not talked about?"
8. Handle any concerns they have, signpost to any local services that could support their concerns.

Kim JS, *et al. BMJ Open* 2024; 14:e078150. doi: 10.1136/bmjopen-2023-078150

An investigation of the modifiable psychosocial variables influencing access to and outcomes after kidney transplantation in children

Interview Topic Guide for Researcher – with NHS professional

Research Goals of the Interview:

7. Explore what the participant thinks matters most to their patients and families in terms of living a good quality life
8. Explore what the participant believes is delaying or enabling families to receive a kidney transplant from a mental health and social factors perspective

[1] Introduction, Overview:

- *Welcome, introduction of the research project and the researcher*
- *Establish a 'stop sign' for research participant to use if needing to pause the interview*

[2] Discussion of…:

Note for Interviewer: Subjects covered in this topic guide are to be explored openly – not leading. Appropriate prompts will be used – e.g. can you tell me more about that? Why do you think that is? How does that make you feel as a professional caring for these families?

7. From their experience of caring for families with ESKD, which aspects of daily-living really matter to these families?
8. What are their experiences of deciding whether a family is ready to prepare for kidney transplantation?
9. What are their experiences of families that do well and not so well after kidney transplantation?

[3] Conclusion:

9. *Summarise what has been discussed, mention the positive details, thank the NHS professional*
10. "How did you find the interview experience?"
11. "Is there anything important to YOU that we have not talked about?"
12. Handle any concerns they have, signpost to any local services that could support their concerns.
