# Supplementary material for: Protocol for a multicentre prospective exploratory mixed-methods study investigating the modifiable psychosocial variables influencing access to and outcomes after kidney transplantation in children and young people in the UK
Source: BMJ Open. 2024 May 28;14(5):e078150. doi: 10.1136/bmjopen-2023-078150 (PMC11138291; doi:10.1136/bmjopen-2023-078150)
Supplement: Supplementary data [file bmjopen-2023-078150supp002.pdf]

Supplementary Material 2

Demographic Background Survey

1. **What is your child's ethnic group?**
   a. White
   b. Black / Black-British
   c. Asian or Asian-British
   d. Mixed ethnicity
   e. Other ethnicity

2. **How many main caregivers (e.g. parents) are in your family?**
   a. One
   b. Two – living in same household
   c. Two – living in separate households
   d. Other ____________

3. **What do you identify yourself as in relation to your child?**
   a. Mother
   b. Father
   c. Aunt
   d. Uncle
   e. Grand mother
   f. Grand father
   g. Foster-carer
   h. Other ____________

4. **What does the other main caregiver identify themselves as in relation to your child? (if applicable)**
   a. Mother
   b. Father
   c. Aunt
   d. Uncle
   e. Grand mother
   f. Grand father
   g. Foster-carer
   h. Other ____________
   i. Not applicable

5. **What is the highest level of education you have completed?**
   a. Primary school
   b. Secondary school up to 16 years
   c. Higher or secondary or further education (e.g. A-levels, IB, BTEC)
   d. College or University
   e. Post-graduate degree

6. **What is the highest level of education, which the other main caregiver has completed? (If applicable)**
   a. Primary school
   b. Secondary school up to 16 years
   c. Higher or secondary or further education (e.g. A-levels, IB, BTEC)
   d. College or University
   e. Post-graduate degree
   f. Not applicable

7. **What is your current employment status?**
   a. Employed – full time
   b. Employed – Part time

Kim JS, *et al. BMJ Open* 2024; 14:e078150. doi: 10.1136/bmjopen-2023-078150

c. Seeking employment
d. Home maker
e. Retired
f. Studying
g. Not seeking employment due to health reasons
h. Not seeking employment due to being full-time carer
i. Not seeking employment due to other reasons ________

8. **What is the other main caregiver's current employment status? (If applicable)**
   a. Employed – full time
   b. Employed – Part time
   c. Seeking employment
   d. Home maker
   e. Retired
   f. Studying
   g. Not seeking employment due to health reasons
   h. Not seeking employment due to being full-time carer
   i. Not seeking employment due to other reasons __________
   j. Not applicable

9. **Is there a recent family history of immigration into the United Kingdom?**
   a. Yes, during my child or their sibling's lifetime (first generation)
   b. Yes, during their parent's lifetime (second generation)
   c. No recent history.

10. **Have you had to move homes after your child was diagnosed with Chronic Kidney Disease?**
    a. Yes, home was inadequate for a child with Chronic Kidney Disease (e.g. unable to store dialysis equipment)
    b. Yes, home was too far from child's hospital where they received care for their kidneys
    c. Yes, but only temporarily (e.g. friend or relative's home or other temporary housing) because ____________
    d. Yes, for other reasons _________________
    e. Not applicable to our family

11. **What is your family's total estimated income in the last year? (before tax, from all sources including salaries and benefits) Chronic Kidney Disease can have a knock-on effect on a family's circumstances, which is why we would like to understand this better by asking this information.**
    a. Up to £10,000
    b. £10,001 to £20,000
    c. £20,001 to £30,000
    d. £30,001 to £50,000
    e. £50,001 to £70,000
    f. £70,001 to £150,000
    g. Over £150,000
    h. Prefer not to say

12. **Next, we would like to ask you questions about your child's medications as this may have an effect on their quality of life.**
    Please select all of the kidney medicines your child is taking at the moment from this menu
    - Aciclovir
    - Azathioprine
    - Calcium Acetate
    - Calcium Carbonate
    - Captopril
    - Cefaclor
    - Cefixim
    - Ciclosporin (Sandimunn)
    - Co-trimoxazole

- Darbepoetin
- Enalapril
- Epoetin
- Everolimus (Certican)
- Ganciclovir
- Growth Hormone
- Irbesartan
- Iron (Ferrous fumarate, Ferrous sulphate)
- Lactulose
- Losartan
- Movicol
- Mycophenolate Mofetil (MMF) (Cellcept, Myfenax)
- Nitrofurantoin
- Potassium Chloride
- Potassium Citrate
- Prednisolone
- Senna
- Sevelamer
- Sirolimus (Rapamune)
- Sodium Bicarbonate
- Sodium Chloride
- Tacrolimus (Advagraf, Prograf, Modigraf)
- Trimethoprim
- Valaciclovir
- Valganciclovir
- Vitamin D (Alfacalcidol)
- Vitamin D (Colecalciferol)
- Other(s)________ ________________

13. Please select how often your child takes these medicines (select all that apply):

a. Longer than every month
b. Every month
c. Every other week
d. Every week
e. Three times a week
f. Every other day
g. Once a day
h. Twice a day
i. Three times a day
j. Four times a day
k. More than four times a day
l. Other regime __________

14. Please select how these medicines are given to your child (select all that apply)

a. An injection directly into their vein (via cannula or other access to their veins)
b. Other type of injection (e.g. into skin or muscle)
c. Tablets (by mouth)
d. Tablets (by gastrostomy tube)
e. Liquids (by mouth)
f. Liquids (by gastrostomy tube)
g. Eye drops
h. Creams / Lotions
i. Inhaler
j. Other type of medicine ____________

Kim JS, *et al. BMJ Open* 2024; 14:e078150. doi: 10.1136/bmjopen-2023-078150

15. **Some families may have had their journey towards a kidney transplant affected by the COVID-19 pandemic. Please select which situation applies to your child and family due to the COVID-19 pandemic in 2020-2021:**
    a. Our family was not in the middle of preparing for a kidney transplant during 2020-2021. Therefore COVID-19 did not impact us.
    b. Our family already started or were starting to prepare for a kidney transplant in 2020-2021. The COVID-19 pandemic did not change our preparation plans.
    c. Our family delayed preparations for a kidney transplant from a living donor due to the COVID-19 pandemic.
    d. Our family came off the waiting list for a kidney transplant from a deceased donor due to the COVID-19 pandemic
    e. Our family delayed investigations that were part of preparing for a kidney transplant overall because of the COVID-19 pandemic
    f. Our family's transplant journey was affected by the COVID-19 pandemic is other ways _________________________
