# Supplementary material for: Protocol for a multicentre prospective exploratory mixed-methods study investigating the modifiable psychosocial variables influencing access to and outcomes after kidney transplantation in children and young people in the UK
Source: BMJ Open. 2024 May 28;14(5):e078150. doi: 10.1136/bmjopen-2023-078150 (PMC11138291; doi:10.1136/bmjopen-2023-078150)
Supplement: Author's manuscript [file bmjopen-2023-078150.draft_revisions.pdf]

BMJ Open is committed to open peer review. As part of this commitment we make the peer review history of every article we publish publicly available.

When an article is published we post the peer reviewers' comments and the authors' responses online. We also post the versions of the paper that were used during peer review. These are the versions that the peer review comments apply to.

The versions of the paper that follow are the versions that were submitted during the peer review process. They are not the versions of record or the final published versions. They should not be cited or distributed as the published version of this manuscript.

BMJ Open is an open access journal and the full, final, typeset and author-corrected version of record of the manuscript is available on our site with no access controls, subscription charges or pay-per-view fees (http://bmjopen.bmj.com).

If you have any questions on BMJ Open's open peer review process please email info.bmjopen@bmj.com

BMJ Open

How to investigate the modifiable psychosocial variables influencing access to and outcomes after kidney transplantation in children and young people

Journal:	*BMJ Open*
Manuscript ID	bmjopen-2023-078150
Article Type:	Protocol
Date Submitted by the Author:	25-Jul-2023
Complete List of Authors:	Kim, Ji Soo; Great Ormond Street Hospital for Children NHS Foundation Trust, Paediatric Nephrology; NIHR Great Ormond Street Hospital Biomedical Research Centre<br>Wray, Jo; Great Ormond Street Hospital for Children NHS Foundation Trust, Centre for Outcomes and Experience Research in Children's Health, Illness and Disability<br>Ridout, Deborah; UCL, Population, Policy and Practice, UCL Great Ormond Street Institute of Child Health<br>Plumb, Lucy ; UK Renal Registry; Bristol Medical School, Population Health Sciences<br>Nitsch, Dorothea; London School of Hygiene & Tropical Medicine; UK Renal Registry<br>Robb, Matthew; NHS Blood and Transplant, Statistics and Clinical Studies<br>Paediatric Nephrology (BAPN), British Association for; British Association for Paediatric Nephrology<br>Marks, Dr. Stephen; Great Ormond Street Hospital For Children NHS Trust, Paediatric Nephrology; NIHR Great Ormond Street Hospital Biomedical Research Centre
Keywords:	Renal transplantation < NEPHROLOGY, Paediatric transplant surgery < PAEDIATRIC SURGERY, Paediatric nephrology < NEPHROLOGY, Health Services Accessibility, Quality of Life, Social Support

SCHOLARONE™
Manuscripts

Note from the Editors: Instructions for reviewers of study protocols

Since launching in 2011, BMJ Open has published study protocols for planned or ongoing research studies. If data collection is complete, we will not consider the manuscript.

Publishing study protocols enables researchers and funding bodies to stay up to date in their fields by providing exposure to research activity that may not otherwise be widely publicised. This can help prevent unnecessary duplication of work and will hopefully enable collaboration. Publishing protocols in full also makes available more information than is currently required by trial registries and increases transparency, making it easier for others (editors, reviewers and readers) to see and understand any deviations from the protocol that occur during the conduct of the study.

The scientific integrity and the credibility of the study data depend substantially on the study design and methodology, which is why the study protocol requires a thorough peer-review.

BMJ Open will consider for publication protocols for any study design, including observational studies and systematic reviews.

Some things to keep in mind when reviewing the study protocol:

- Protocol papers should report planned or ongoing studies. The dates of the study should be included in the manuscript.
- Unfortunately we are unable to customize the reviewer report form for study protocols. As such, some of the items (i.e., those pertaining to results) on the form should be scores as Not Applicable (N/A).
- While some baseline data can be presented, there should be no results or conclusions present in the study protocol.
- For studies that are ongoing, it is generally the case that very few changes can be made to the methodology. As such, requests for revisions are generally clarifications for the rationale or details relating to the methods. If there is a major flaw in the study that would prevent a sound interpretation of the data, we would expect the study protocol to be rejected.

For peer review only

How to investigate the modifiable psychosocial variables influencing access to and outcomes after kidney transplantation in children and young people

Ji Soo Kim[1,2], Jo Wray[3,] Deborah Ridout[4], Lucy Plumb[5,6], Dorothea Nitsch[6,7], Matthew Robb[8], British Association for Paediatric Nephrology, Stephen D Marks[1,2]

For peer review only

1. Department of Paediatric Nephrology, Great Ormond Street Hospital for Children NHS Foundation Trust, Great Ormond Street London, UK
2. NIHR Great Ormond Street Hospital Biomedical Research Centre, University College London Great Ormond Street Institute of Child Health, London, UK
3. Centre for Outcomes and Experience Research in Children's Health, Illness and Disability, Great Ormond Street Hospital for Children NHS Foundation Trust, London, UK
4. Population, Policy and Practice Department, University College London Great Ormond Street Institute of Child Health, London, UK
5. Population Health Sciences, University of Bristol Medical School, Bristol, UK.
6. UK Renal Registry, Bristol, UK
7. Department of Non-communicable disease epidemiology, London School of Hygiene & Tropical Medicine
8. Statistics and Clinical Studies team at NHS Blood and Transplant, Bristol, UK

Address for Correspondence:
Professor Stephen D Marks, Dr Ji Soo Kim
Department of Paediatric Nephrology
Great Ormond Street Hospital for Children NHS Foundation Trust
London
WC1N 3JH
stephen.marks@gosh.nhs.uk jisoo.kim@nhs.net

Word Count: 3662/4000 words (excluding title page, abstract, tables, references)
Abstract: 296/300 words

For peer review only - http://bmjopen.bmj.com/site/about/guidelines.xhtml

Abbreviations

BAPN – British Association for Paediatric Nephrology

CKD-5 – Stage V chronic kidney disease

CYP – children and young people

KRT – kidney replacement therapy

MDT – multi-disciplinary team

NIHR – National Institute for Health Research

QoL – Quality of Life

RPF – Research Partner Family

SG – steering group

YPAG – Young Persons Advisory Group

Keywords

Renal transplantation

Paediatric transplant surgery

Paediatric nephrology

Health Services Accessibility

Quality of Life

Social Support

For peer review only

For peer review only - http://bmjopen.bmj.com/site/about/guidelines.xhtml

Abstract

Introduction

Kidney transplantation is the preferred therapy for children with Stage 5 chronic kidney disease (CKD-5). However, there is wide variation in access to kidney transplantation across the UK for children. This study aims to explore the psychosocial factors that influence access to and outcomes after kidney transplantation in children in the UK using a mixed methods prospective longitudinal design.

Methods

Qualitative data will be collected through semi-structured interviews with children affected by CKD-5, their carers and their paediatric renal multi-disciplinary team. These interviews will inform the choice of existing validated questionnaires for the second phase of the study, during which selected questionnaires will be distributed to a larger national cohort of children with pre-transplant CKD-5 and their carers. Follow-up questionnaires will be sent at protocolised time points regardless of whether they receive a kidney transplant or not. Co-existing health data from hospital, UK renal registry and NHS Blood and Transplant registry records will be mapped to each questionnaire time point. An integrative analysis of the mixed qualitative and quantitative data will define psychosocial aspects of care for potential intervention to improve transplant access.

Analysis

Qualitative data will be analysed using thematic analysis. Quantitative data will be analysed using appropriate statistical methods to understand how these factors influence access to transplantation, as well as the distribution of psychosocial factors pre- and post-transplantation.

Ethics and Dissemination

This study protocol has been reviewed by the NIHR Academy and approved by the Wales Research Ethics Committee 4 (IRAS number 270493/ref: 20/WA/0285) and the Scotland A Research Ethics Committee (ref: 21/SS/0038). Results from this study will be disseminated across

For peer review only

media platforms accessed by affected families, presented at conferences, and published in peer-reviewed journals.

Registration Details

This work is supported by the National Institute for Health Research Academy, grant number NIHR300727.

For peer review only

For peer review only - http://bmjopen.bmj.com/site/about/guidelines.xhtml

Article Summary:

Strengths and limitations of this study

1. Prospective, longitudinal study design allows for a more detailed understanding of delays in transplantation associated with different psychosocial factors.
2. Combination of qualitative and quantitative data enable in-depth exploration of how psychosocial factors influence access to transplantation and outcomes thereafter.
3. Involvement of the majority of UK paediatric nephrology units ensuring representation of paediatric CKD-5 population
4. Limited follow-up timeline will capture only short to medium-term rather than long-term outcomes.

For peer review only

For peer review only - http://bmjopen.bmj.com/site/about/guidelines.xhtml

Introduction

Around 1,000 children and young people (CYP) with Stage 5 chronic kidney disease (CKD-5) in the United Kingdom receive Kidney Replacement Therapy (KRT) in the form of either peritoneal dialysis, haemodialysis or kidney transplantation[1, 2]. Kidney transplantation is the gold standard therapy for reducing mortality and improving outcomes for CYP[3]. Minimising time on dialysis in favour of transplantation has been shown to reduce CKD-5 related complications and morbidity [4]. Compared with dialysis, transplantation is also presumed to improve patients' health-related quality of life (HR-QoL)[5]. Furthermore in the UK, for every year that the patient's renal allograft continues to function, transplantation is three times more cost-effective than dialysis for the NHS[6].

However, not every CYP with CKD-5 is able to access kidney transplantation. There are approximately 193 to 217 prevalent CYP on dialysis each year[7, 8]. Annually only 130-160 paediatric kidney transplants are performed in the UK[9]. There appears to be variation in transplantation access, practice and outcomes between all 13 UK paediatric nephrology units[1, 2, 9]. A cross-sectional survey was conducted by the British Association for Paediatric Nephrology (BAPN) to examine reasons for paediatric kidney transplantation delay in the UK. The survey has shown that psychosocial factors make up 19% of the barriers, although specific factors were not identified as part of this study[10]. Compared with paediatrics, considerable research has been done in adult patients, regarding psychosocial factors implicated in transplant access. Formalised pre- and post-transplant psychosocial assessments have been widely researched for adult solid organ transplant recipients[11-14]. The UK-wide study 'Access to Transplantation and Transplant Outcome Measures' specifically investigated psychosocial barriers in adult kidney transplant recipients[15]. Researchers found inequities in transplant access, in spite of a universal healthcare system, based on socioeconomic status, education level, health literacy and racial background[16, 17]. In terms of outcomes, they found no difference in post-transplant HR-QoL between living and deceased donor recipients and that recipient expectations influenced post-transplant recovery[15-19]. However, in paediatrics, it is less clear what these 'psychosocial factors' are, let alone how

For peer review only - http://bmjopen.bmj.com/site/about/guidelines.xhtml

they influence unit-specific decisions, access to kidney transplantation and their outcomes in the UK[10, 20, 21]. To date, psychosocial studies for CYP with CKD-5 are limited by retrospective or cross-sectional design or by small study cohorts. There are no studies that prospectively explore how these factors impact kidney transplantation access.

This study aims to prospectively evaluate the psychosocial factors that are actual or perceived barriers to paediatric kidney transplantation which may be associated with poor transplant outcome. We hypothesise that psychosocial factors that are implicated in transplant access can be quantified using formal, validated measures, and that these factors may influence outcomes in the short-term period following transplantation.

This will be achieved through the following research objectives:

1. Describe the psychosocial factors perceived by clinicians as barriers to kidney transplantation.
2. Describe current interventions (if any) implemented to address these psychosocial factors.
3. Explore the experiences and beliefs of CYP and their families of any psychosocial challenges or facilitators in accessing a kidney transplant
4. Quantify the identified psychosocial factors implicated in CKD management
5. Measure the prevalence of identified psychosocial factors (positive and negative) in the national cohort of patients being pre-emptively worked up for transplant, on dialysis or listed for transplant.
6. Examine these psychosocial factors regarding their association with time to transplant and their changes following transplantation
7. Synthesise findings from each phase to inform recommendations about which psychosocial factors are potential barriers or facilitators to accessing kidney transplantation.

For peer review only - http://bmjopen.bmj.com/site/about/guidelines.xhtml

Findings synthesised from this study will inform development of a complex intervention to improve uptake of kidney transplantation in CYP with CKD-5 who would benefit most from one.

Methods and Analysis

Study Design Overview:

This prospective study has three phases, commencing with a sequential exploratory mixed-methods design, followed by a sequential explanatory mixed-methods design (Figure 1)[22]. This approach was chosen to first gain new insights by becoming familiar with the range of potential psychosocial factors and then longitudinally observing the influence of these factors on kidney transplantation access and outcomes for CYP[23]. Phase one will consist of exploratory interviews with purposively selected participants. These interviews will inform which questionnaires will be distributed to the wider cohort of CYP with CKD-5 and their carer(s) in phase two stage A. Participant families with outlier findings from phase two stage A will then be invited for explanatory interviews in phase two stage B. Finally, in phase three, the qualitative and quantitative data from the previous phases will be analysed together to develop an integrated understanding of psychosocial factors that influence access to kidney transplantation. All elements of the study design were co-developed and approved by our Research Partner Family (RPF) and study steering group (SG). Our RPF (a young person and parent dyad) and SG members have lived experience of CKD, dialysis and kidney transplantation in childhood either as a patient or carer. The Great Ormond Street Hospital Young Persons Advisory Group (GOSH YPAG) have also approved the research question and study design. The GOSH YPAG is a group of CYP affiliated with Great Ormond Street Hospital for Children NHS Foundation Trust as a patient or family member of a patient and therefore who have the lived experience needed to advise researchers designing studies involving children.

For peer review only - http://bmjopen.bmj.com/site/about/guidelines.xhtml

Population:

This study aims to include CYP, carer and staff participants from all 13 UK paediatric nephrology units, of which 10 units offer kidney transplantation surgery on site and 3 units which do not offer transplantation surgery at their own site but offer shared transplant care with nearby units.

In phase one and phase two stage A, CYP (aged 0 – 17 years inclusive) with CKD-5 on chronic dialysis, being worked up for pre-emptive kidney transplantation or on the waiting list for deceased donation or awaiting living donation, will be invited to participate with their carer(s). Members of the multi-disciplinary team (MDT) involved in pre-transplantation work-up at their local unit (e.g., paediatric nephrologists, transplant surgeons, nurse specialists, social workers, family therapists, play therapists and members of the psychosocial team etc.) will be invited for interviews in Phase One only. Patients or carer(s) who are unable to give informed consent or patients who have been deemed too unwell to participate or had a recent acute hospital admission in the last 14 days will not be approached to participate in the study.

Phase One Interviews:

A purposive sampling matrix will be used to maximise the diversity of views. Criteria for sampling will include age, sex, and ethnicity of CYP, modality of KRT, whether the kidney unit offers transplantation surgery or not and role in the MDT. Completed interviews will be analysed in parallel with ongoing recruitment. Participant recruitment will stop when no new themes are generated from the data.

Analysis of these interviews will contribute towards selecting questionnaires for use in phase two stage A.

For peer review only - http://bmjopen.bmj.com/site/about/guidelines.xhtml

Phase Two Stage A Questionnaires:

In this phase, we will aim to recruit every CYP with CKD-5 who meets the inclusion criteria at pre-transplant baseline with an anticipated recruitment rate of 60-70%.

At the time of study design, the predicted annual numbers across all 13 UK paediatric nephrology units as per UK Renal Registry (UKRR) reports were as follows[1, 2]:

- Number of dialysis patients on the transplant waiting-list in a year; n=70
- Number of patients starting dialysis in a year; n=125
- Number of patients not on dialysis but pre-emptively on the transplant waiting list; n=50
- Transplant rate; 130 to 160 patients per year

Based on these numbers, the predicted maximum number of eligible participants in a 1-year-period would be 245. We chose a 2-year-period for our sample size calculation, which would be a population size of 490, to include CYP who experience delays and receive their transplant after 1 year (See Figure 2). We plan to recruit 180 patients and after allowing for a 20% drop out, we expect to include 141 families. Assuming a prevalence of pertinent psychosocial factors in the national cohort of patients being pre-emptively worked up for transplant, on dialysis or listed for transplant of 15%, then with 141 families we will be able to estimate this with 5% precision. At the time of sample size calculation, there were no paediatric studies specifically measuring this nor studies encompassing all psychosocial factors. Therefore, we used the assumed prevalence of 15% based on a study measuring psychological distress in potential adult transplant candidates[24].

Phase Two Stage B:

Results from phase two stage A will be reviewed for negative cases. Families who have outlier findings, in terms of their answers to the validated questionnaires, will be invited to interview in phase two stage B, with the aim of reviewing and refining themes to develop additional theoretical explanation to the influence of psychosocial factors on transplant access and outcomes. Exact

For peer review only - http://bmjopen.bmj.com/site/about/guidelines.xhtml

parameters to define outliers and sample size will be dependent on findings from phase two stage A.

Outcomes measured:

Phase One:

Using a Topic Guide (Supplementary Material 1), qualitative data will be collected in phase one to address research objectives 1-4 through semi-structured, in-depth interviews undertaken by the investigator (JSK). The family interview format will be based on the CYP participants' decision to either interview with or separately from their carers, depending on which setting they find more comfortable. The research goals for family interviews will include exploring what families feel their life is like now living with CKD-5, what good HR-QoL looks like and what they believe delays or enables how soon they receive a kidney transplant. Similarly, research goals for MDT member interviews will include exploring what the professionals think matters most to families whose CYP has CKD-5 when it comes to a good HR-QoL and what they, as professionals, believe impacts how soon CYP access a kidney transplant in terms of psychosocial factors.

The Topic Guide has been developed from the investigator's systematic literature review on the subject matter and in consultation with the GOSH YPAG and the RPF[25]. To minimise any inconvenience for joining the study, all participants will be interviewed using their preferred modality (telephone, video-call or face-to-face consultations) in keeping with COVID-19 safety recommendations[26]. Interview time with young children will be kept to less than half an hour to minimise interview fatigue. All interviews will be audio or video recorded depending on participant preference.

The acceptability of existing validated age-appropriate questionnaires that measure outcomes relevant to the preliminary themes, will be discussed with the SG. Potential validated questionnaires that capture the preliminary themes will be identified from the systematic literature review and a wider search of the literature. These questionnaires will be checked in terms of their

For peer review only - http://bmjopen.bmj.com/site/about/guidelines.xhtml

psychometric properties such as internal consistency (Cronbach alpha of at least 0.7) and test re-test reliability and aspects such as availability of the measures, respondent type (parent, child, or other respondent) and age range for which the questionnaire has been validated, to enable the most appropriate questionnaire to be chosen to measure each theme. The selected questionnaires will then be discussed with the SG, considering the language of the questionnaire, acceptability, and level of burden for the participant. If the list of questionnaires is too onerous for the participating family, a consensus will be reached with the SG on which preliminary themes and therefore which questionnaires should be prioritised. Once the final list of questionnaires is agreed on, these will be submitted to the Health Research Authority for final approval.

Phase Two Stage A:

In phase two stage A, research objectives 5-6 will be addressed by measuring the following primary outcome variables: HR-QoL, psychosocial functioning and time taken to receive a kidney transplant since date confirming CKD-5. To understand changes in psychosocial factors over time, their associations with health burden or short-term allograft deterioration must be accounted for, Therefore, we will measure the following secondary outcome: the CYP's estimated glomerular filtration rate (eGFR) over time. The eGFR will be calculated with their height and serum creatinine using the CkiD U25 formula [27, 28].

Questionnaires measuring HR-QoL and psychosocial functioning, selected from phase one, will be distributed to a larger, national cohort of CYP with CKD-5 and their carers. Follow-up questionnaires will be sent post-transplant at 3-, 6- and 12-months later or 12-months after their first questionnaire if they still have not received a kidney transplant in that time frame. These follow-up time points were chosen to reflect the initial period of post-transplant adaptation, which are comparable with similar studies in adults and children at the time of protocol-writing[29-31]. As families are more likely to participate if they can choose which questionnaire modality is most suited to their lifestyle, participant families will be offered either paper or online questionnaires[32].

For peer review only - http://bmjopen.bmj.com/site/about/guidelines.xhtml

Health morbidity and co-existing disease data, including their underlying primary kidney diagnosis, as described in Table 1, will be mapped to each questionnaire time point.

An additional questionnaire (see Supplementary Material 2) has been co-designed with the SG to retrieve information on the participating family's demographic background. Data on medication burden will also be collected due to its' possible confounding effect HR-QoL as described in current literature[33].

Phase Two Stage B:

The Interview Topic Guide for Phase Two Stage B has been designed with flexibility, since the interview will depend on the nature of the outlier findings from Phase Two Stage A.

Data Analysis Plan:

Phase One:

Interview transcripts will be thematically analysed, following the approach of Braun and Clarke [34], by JSK. De-identified transcripts will be managed using Nvivo software[35]. For the purposes of qualitative rigour, JSK will maintain a reflexivity journal after interviews and throughout data analysis, to ensure potential researcher biases and insights are noted. Maintaining a reflexivity journal is a gold standard practice in qualitative research, which increases the credibility and deepens the understanding of the findings by describing the context in which data were collected and analysed[36, 37]. A sample of transcripts will be examined by JW and discussed with JSK. Throughout all stages of data collection and analysis, findings will be shared and discussed by JSK, JW and SDM.

For peer review only - http://bmjopen.bmj.com/site/about/guidelines.xhtml

Phase Two Stage A:

First, descriptive statistics will be used to describe participant demographics, prevalence of psychosocial factors and how participants' psychosocial functioning and HR-QoL variables change before and after transplantation at each data collection time point.

Second, to describe the impact waiting to access a kidney transplant and receiving one has on psychosocial functioning and HR-QoL over time, repeated measure ANOVA will be undertaken.

Third, the level of agreement between CYP and carer responses will be assessed. Choice of statistical tests will depend on the type of data: for example, Cohen's Kappa for binary data, weighted Kappa for ordinal data or interclass correlation coefficients for continuous data.

Fourth, the association between clinical, demographic, and psychosocial factors with accessing a kidney transplant will be measured using logistic regression modelling and Kaplan-Meier statistics.

Finally, the association of clinical, demographic, and psychosocial factors with failing transplant allograft in short-term follow-up between one- and two-years post-transplantation will be assessed. Where the outcome variable is the CYP's eGFR, linear regression modelling will be used. Where the outcome variable defines a failing post-transplant kidney as reaching an eGFR equivalent to stage IV CKD (eGFR 15-29 ml/min/1.73m^2), logistic regression modelling and Kaplan-Meier statistics will be utilised.

Where relevant, all statistical analyses will be adjusted for (but not limited to) CYP's age, gender, ethnicity, socioeconomic status, and KRT status at baseline.

Phase Two Stage B:

Interview data will be analysed using Thematic Analysis underpinned by the same principles as in phase one.

For peer review only - http://bmjopen.bmj.com/site/about/guidelines.xhtml

Phase Three:

An integrative analysis of data from phases one and two will be undertaken to create a conceptual model of how psychosocial factors influence transplant access and outcomes. The quantitative and qualitative data will be reviewed together to understand where they converge, complement, or diverge from the other dataset or have findings that are novel. A final lay report will be co-written with the SG to ensure it is meaningful, relevant, and accessible to families whose CYP have CKD.

Ethics

This study protocol has been peer reviewed by the National Institute for Health Research (NIHR) Academy and has been approved under IRAS number 270493 by the Wales Research Ethics Committee 4 (ref: 20/WA/0285) and the Scotland A Research Ethics Committee (ref: 21/SS/0038).

Patient and public involvement, engagement and participation is integral to ensuring that all aspects of study procedure and materials are ethically sound and acceptable to participant families and CYP of different ages. As aforementioned, the study design and all participant-facing materials were discussed with the GOSH YPAG, the RPF and the SG.

Coercive pressure of joining the study will be minimised. Research participants will not be paid for participation. Participant information sheets will indicate that there will be no added benefit or disruption to their medical care.

Children will be encouraged to be an active participant by obtaining Informed written consent from all participants aged ≥16 years old and written assent from participants aged 5 – 15 years old. Consent will be obtained for all research activities including interviews, questionnaires and retrieving health information from national databases and hospital records.

To ensure no participant is unfairly excluded in phase one interviews due to English not being their first language, interpreters will be present to support their participation. The same principle of

For peer review only

minimising communication barriers will be applied for younger children or young people who may prefer communicating through other creative outputs such as drawing or Talking Mats (ref).

Some CYP may prefer their carers to be present to help express themselves more fully whereas some may prefer their carers to be absent to express themselves more freely. Therefore, all CYP will be given the option to be interviewed with or without their carer in the room.

Participants in the phase one interviews may become distressed as they reflect on their experiences due to the sensitive nature of some of the interview topics around their mental health or transplant delays. Therefore, the participant and JSK will agree on a 'stop signal' before commencing the interview for use should they feel uncomfortable. If the participant uses the 'stop signal', they will be offered a break. The participant and JSK will then discuss whether they would like to continue, reschedule or withdraw from the interview altogether. Once the interview recording stops, there will be an opportunity to discuss the participant's feelings and if appropriate, they will be signposted to their local support services.

Participant confidentiality will be upheld by fully adhering to the Data Protection Act. Participant identifiers will be handled with appropriate pseudonymisation and all data will be kept on GDPR-compliant encrypted devices or stored on hard drives with restricted access with the relevant encryption and password protection. The only instance where confidentiality will be breached is if a participant discloses information that has direct implications for child or adult safeguarding. Potential participants will be made aware of this as part of informed consent. All interviews will be digitally recorded, transcribed verbatim and have identifiable data redacted. Audio files will be transcribed either by JSK or by a third-party interview transcription company (Take Note®). To minimise data handling breaches, all engagement with Take Note® will only be through their secure web platform. All video files will be transcribed only by JSK to remove third party involvement in the de-identification process. Once de-identified, only quotes that cannot lead to participant identification will be used in reports. Care will be taken in reporting findings to ensure individuals cannot be identified by their role, diagnosis, gender, age or geographic locality.

For peer review only - http://bmjopen.bmj.com/site/about/guidelines.xhtml

Dissemination

Given the relevance this project has to both patient families and their clinicians, a targeted dissemination strategy will be adopted. Research participants will be updated about the findings through newsletters. Lay summaries approved by the SG will be disseminated across charity websites accessed by CYP and families affected by CKD. To ensure that professionals who work with families affected by CKD are being reached, findings will be disseminated widely in relevant peer-reviewed journals and at national and international conferences. Finally, if appropriate, a dissemination strategy will be co-created between JSK and the SG for other professionals (e.g., teachers) who may come into contact with vulnerable families with CKD who may need early referral for psychosocial intervention.

In conclusion, psychosocial factors influencing access to and outcomes after paediatric kidney transplantation are poorly understood. Given that almost one fifth of eligible CYP are delayed from receiving kidney transplantation due to psychosocial factors, this is an important area of study[10]. The mixed-methods, prospective, and longitudinal design of this research project, has the potential to advance our understanding of how these factors truly influence access to transplantation in a way that a retrospective or cross-sectional study cannot. Findings from this project will be able to be utilised by UK paediatric nephrology units to optimise and standardise their psychosocial care for CYP being prepared for kidney transplantation. Finally, findings will also inform future research into developing interventions that better target aspects of psychosocial support for CYP and their families to improve their access to and short-term outcomes of kidney transplantation.

For peer review only - http://bmjopen.bmj.com/site/about/guidelines.xhtml

Author's contribution

- JSK wrote the study protocol and coordinated the entire manuscript.
- Deborah Ridout contributed towards sample size calculation and statistical methods of study protocol.
- LP contributed towards statistical analysis of protocol and advised regarding UK Renal Registry involvement.
- DN contributed towards statistical analysis of protocol and advised regarding UK Renal Registry involvement.
- MR contributed towards statistical analysis of protocol and advised regarding NHS Blood & Transplant involvement.
- JW supervised JSK, study protocol development with regular input to initial drafts and final manuscript approval.
- SDM supervised JSK, study protocol development with regular input to initial drafts and final manuscript approval.

For peer review only

For peer review only - http://bmjopen.bmj.com/site/about/guidelines.xhtml

Acknowledgements

We would like to thank the Great Ormond Street Hospital Young Person's Advisory Group and members of our Research Partner Family Steering Group –Emma Beeden, Katy Beeden, Angela Watt and Heather Davis from KDARS (Kidney Disease and Renal Support) for kids, for their expertise, support and advice. Finally, we would also like to thank members of the British Association for Paediatric Nephrology, the National Kidney Federation and Kidney Care UK for their continued support of this study.

Funding Statement:

This work is supported by the National Institute for Health Research Academy, as a Doctoral Fellowship, grant number NIHR300727.

Competing Interests Statement:

Dr Ji Soo Kim is the NIHR Fellowship grant recipient, which funds this study.

Dr Lucy Plumb reports grants from NIHR and Kidney Research UK. She is also the paediatric research lead for the UK Renal Registry.

For peer review only

References

1. Plumb L, Casula A, Pyart R, Evans KM, Inward C, Medcalf J, et al. The 21st UK Renal Registry Annual Report: A Summary of Analyses of Paediatric Data in 2017. Nephron. 2019:1-5.
2. Plumb L, Wong E, Casula A, Braddon F, Lewis M, Marks SD, et al. Chapter 4 Demography of the UK Paediatric Renal Replacement Therapy Population in 2016. Nephron. 2018;139 Suppl 1:105-16.
3. Wolfe RA, Ashby VB, Milford EL, Ojo AO, Ettenger RE, Agodoa LYC, et al. Comparison of Mortality in All Patients on Dialysis, Patients on Dialysis Awaiting Transplantation, and Recipients of a First Cadaveric Transplant. New England Journal of Medicine. 1999;341(23):1725-30.
4. Pruthi R, Casula A, Inward C, Roderick P, Sinha MD. Early Requirement for RRT in Children at Presentation in the United Kingdom: Association with Transplantation and Survival. Clin J Am Soc Nephrol. 2016;11(5):795-802.
5. Park KS, Hwang Yj Fau - Cho MH, Cho Mh Fau - Ko CW, Ko Cw Fau - Ha IS, Ha Is Fau - Kang HG, Kang Hg Fau - Cheong HI, et al. Quality of life in children with end-stage renal disease based on a PedsQL ESRD module. 2012(1432-198X (Electronic)).
6. Kerr M, Bray B, Medcalf J, O'Donoghue DJ, Matthews B. Estimating the financial cost of chronic kidney disease to the NHS in England. Nephrol Dial Transplant. 2012;27 Suppl 3:iii73-80.
7. UKRR. UK Renal Registry 21st Annual Report - data to 31/12/2017 Bristol, UK2019 [cited 2023. Available from: https://www.renalreg.org/publications-reports/.
8. UKRR. UK Renal Registry 24th Annual Report - data to 31/12/2020 Bristol, UK2022 [Available from: https://ukkidney.org/audit-research/annual-report.
9. Transplant OD. Annual Report on Kidney Transplantation Report for 2016/2017 NHS Blood and Transplant2017 [Available from: https://nhsbtdbe.blob.core.windows.net/umbraco-assets-corp/4607/kidney-annual-report-2016-17.pdf.
10. Kim JS, Marlais M, Balasubramanian R, Muorah M, Inward C, Smith GC, et al. UK national study of barriers to renal transplantation in children. Archives of disease in childhood. 2020.
11. Feurer ID, Moore DE, Speroff T, Liu H, Payne J, Harrison C, et al. Refining a health-related quality of life assessment strategy for solid organ transplant patients. Clin Transplant. 2004;18 Suppl 12:39-45.
12. Goetzmann L, Ruegg L, Stamm M, Ambuhl P, Boehler A, Halter J, et al. Psychosocial profiles after transplantation: a 24-month follow-up of heart, lung, liver, kidney and allogeneic bone-marrow patients. Transplantation. 2008;86(5):662-8.
13. Maldonado JR, Sher Y, Lolak S, Swendsen H, Skibola D, Neri E, et al. The Stanford Integrated Psychosocial Assessment for Transplantation: A Prospective Study of Medical and Psychosocial Outcomes. Psychosom Med. 2015;77(9):1018-30.
14. Shah VS, Ananth A, Sohal GK, Bertges-Yost W, Eshelman A, Parasuraman RK, et al. Quality of life and psychosocial factors in renal transplant recipients. Transplant Proc. 2006;38(5):1283-5.
15. Wu DA, Robb ML, Watson CJE, Forsythe JLR, Tomson CRV, Cairns J, et al. Barriers to living donor kidney transplantation in the United Kingdom: a national observational study. Nephrol Dial Transplant. 2017;32(5):890-900.
16. Pruthi R, Robb ML, Oniscu GC, Tomson C, Bradley A, Forsythe JL, et al. Inequity in Access to Transplantation in the United Kingdom. Clin J Am Soc Nephrol. 2020;15(6):830-42.
17. Taylor DM, Bradley JA, Bradley C, Draper H, Dudley C, Fogarty D, et al. Limited health literacy is associated with reduced access to kidney transplantation. Kidney Int. 2019;95(5):1244-52.
18. Wu DA, Robb ML, Forsythe JLR, Bradley C, Cairns J, Draper H, et al. Recipient Comorbidity and Survival Outcomes after Kidney Transplantation: a UK-wide Prospective Cohort Study. Transplantation. 2019.

For peer review only - http://bmjopen.bmj.com/site/about/guidelines.xhtml

19. Gibbons A, Bayfield J, Cinnirella M, Draper H, Johnson RJ, Oniscu GC, et al. Changes in quality of life (QoL) and other patient-reported outcome measures (PROMs) in living-donor and deceased-donor kidney transplant recipients and those awaiting transplantation in the UK ATTOM programme: a longitudinal cohort questionnaire survey with additional qualitative interviews. BMJ Open. 2021;11(4):e047263.
20. Fung E, Shaw RJ. Pediatric Transplant Rating Instrument - a scale for the pretransplant psychiatric evaluation of pediatric organ transplant recipients. Pediatr Transplant. 2008;12(1):57-66.
21. Killian MO, Schuman DL, Mayersohn GS, Triplett KN. Psychosocial predictors of medication non-adherence in pediatric organ transplantation: A systematic review. Pediatr Transplant. 2018;22(4):e13188.
22. Creswell JW. A Concise Introduction to Mixed Methods Research. 1 ed. Thousand Oaks, United States: SAGE Publications Inc; 2014.
23. Reiter B. Theory and Methodology of Exploratory Social Science Research. International Journal of Science and Research Methodology 2017;5(4):129-50.
24. Kuntz KK, Bonfiglio DB. Psychological distress in patients presenting for initial renal transplant evaluation. J Clin Psychol Med Settings. 2011;18(3):307-11.
25. Kim J, Chesters H, Wray J, Marks SD. What are the psychosocial factors that influence access to kidney transplantation (and their outcomes) for children and young people? A Systematic Literature Review Protocol University of York: PROSPERO; 2020 [cited 2021. Available from: https://www.crd.york.ac.uk/PROSPEROFILES/210506_PROTOCOL_20210413.pdf.
26. BAPN. COVID-19 Updated Shielding Guidance for Children and Young People with Kidney Disease UK: Renal Association; 2020 [cited 2020. Available from: https://renal.org/wp-content/uploads/2020/06/Shielding-guidance-25.06.2020.pdf.
27. Ng DK, Pierce CB. Kidney Disease Progression in Children and Young Adults With Pediatric CKD: Epidemiologic Perspectives and Clinical Applications. Semin Nephrol. 2021;41(5):405-15.
28. Pierce CB, Muñoz A, Ng DK, Warady BA, Furth SL, Schwartz GJ. Age- and sex-dependent clinical equations to estimate glomerular filtration rates in children and young adults with chronic kidney disease. Kidney Int. 2021;99(4):948-56.
29. Kugler C, Gottlieb J, Warnecke G, Schwarz A, Weissenborn K, Barg-Hock H, et al. Health-related quality of life after solid organ transplantation: a prospective, multiorgan cohort study. Transplantation. 2013;96(3):316-23.
30. Wightman A, Bradford MC, Smith J. Health-related quality of life changes following renal transplantation in children. Pediatr Transplant. 2019;23(2):e13333.
31. Wyld M, Morton RL, Hayen A, Howard K, Webster AC. A systematic review and meta-analysis of utility-based quality of life in chronic kidney disease treatments. PLoS Med. 2012;9(9):e1001307.
32. Pulham RA, Wray J, Feinstein Y, Brown K, Pierce C, Nadel S, et al. Feasibility and Acceptability of Methods to Collect Follow-Up Information From Parents 12 Months After Their Child's Emergency Admission to Pediatric Intensive Care. Pediatr Crit Care Med. 2019;20(4):e199-e207.
33. Díaz-González de Ferris ME, Pierce CB, Gipson DS, Furth SL, Warady BA, Hooper SR. Health-related quality of life in children with chronic kidney disease is affected by the number of medications. Pediatr Nephrol. 2021;36(5):1307-10.
34. Braun V, Clarke V. Using thematic analysis in psychology. Qualitative Research in Psychology. 2006;3(2):77-101.
35. Lumivero. NVivo Denver2020 [cited 2023 2023]. 2020:[Available from: https://lumivero.com/products/nvivo/.
36. Dodgson JE. Reflexivity in Qualitative Research. J Hum Lact. 2019;35(2):220-2.
37. Russell GM, Kelly NH. Research as Interacting Dialogic Processes: Implications for Reflexivity. Forum Qualitative Sozialforschung / Forum: Qualitative Social Research. 2002;3(3).

For peer review only - http://bmjopen.bmj.com/site/about/guidelines.xhtml

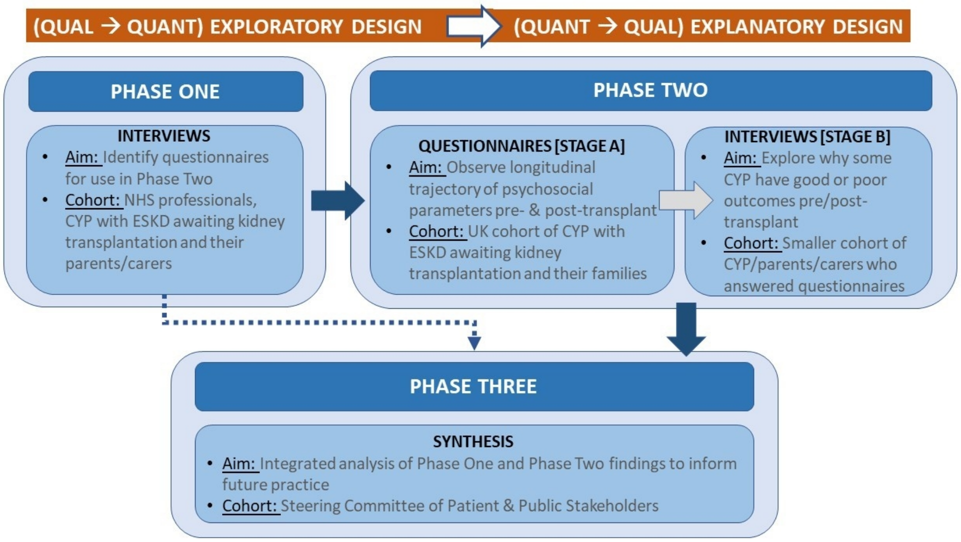

Flow diagram of Mixed Methods study design

338x190mm (96 x 96 DPI)

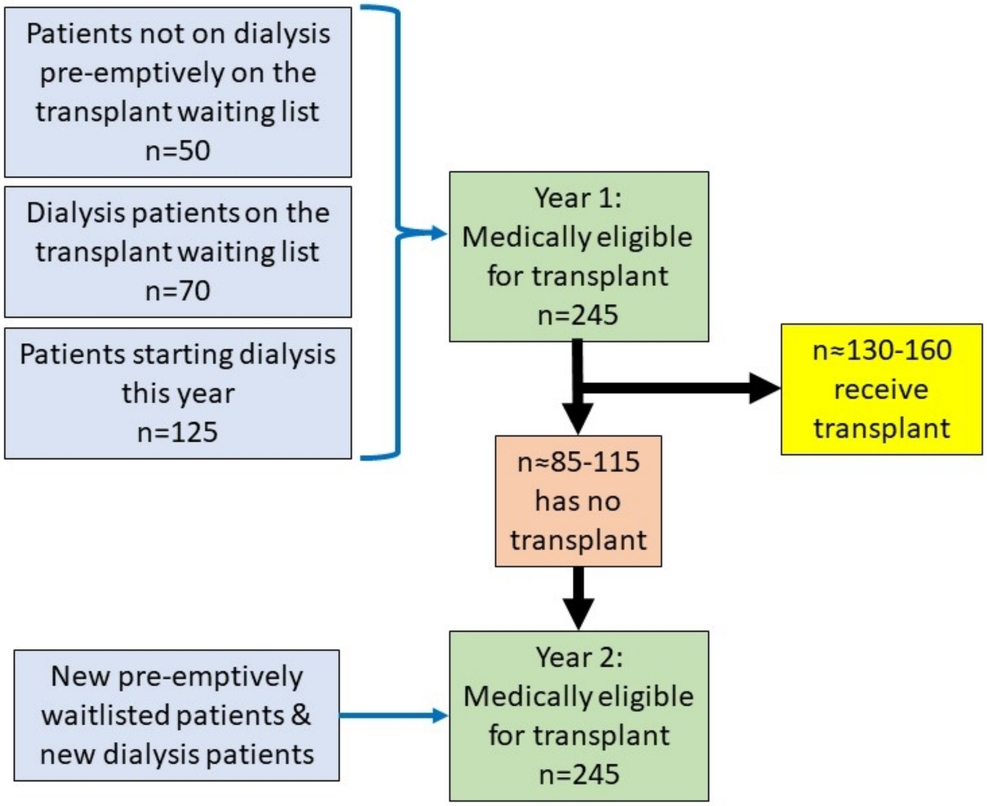

Flow diagram illustrating predicted number of CYP who are eligible for transplantation and receive one across two years

196x159mm (96 x 96 DPI)

Baseline of all participating CYP	CYP not transplanted yet at 12 months follow-up	CYP who are Post-transplant
• Ethnicity<br>• Primary renal diagnosis<br>• Other co-morbidity diagnoses<br>• Modality of KRT if applicable<br>• Date confirming CKD-5<br>• Date of starting RRT if applicable<br>• Date of previous kidney transplant reaching CKD-5 if applicable<br>• Height (cm)<br>• Weight (kg)<br>• Serum Creatinine (ummol/L)<br>• Number and type of medications*	• Modality of KRT if changed (if applicable)<br>• Date of starting new modality of RRT (if applicable)<br>• Height (cm)<br>• Weight (kg)<br>• Serum Creatinine (ummol/L)<br>• Number and type of medications*	At 3 months post-transplant<br>• Type of transplant kidney (Living related / Living Unrelated / Deceased Donor (after cardiac or brain death)<br>• Donor & recipient mismatch<br>• ABO compatibility<br>• Cold ischaemic Time<br>• Need for plasma exchange / immunoadsorption / Rituximab / iv immunoglobulin<br>At 6-, 12- and 24-months post-transplant<br>• Changes to co-morbidities if applicable<br>• Change of KRT modality if applicable<br>• Date of RRT modality change if applicable<br>• Height (cm)<br>• Weight (kg)<br>• Serum Creatinine (ummol/L)<br>• Number and type of medications*

Table 1: Co-existing Health Data to be collected and mapped against questionnaire time points

For peer review only

Supplementary Material 1

An investigation of the modifiable psychosocial variables influencing access to and outcomes after kidney transplantation in children

Interview Topic Guide for Researcher – with parent / guardian

Research Goals of the Interview:

1. Explore how the participant feels their life is now (with End Stage Kidney Disease [ESKD])
2. Explore what good quality life looks like to the participant – what matters most?
3. Explore what the participant believes is delaying or enabling their child to receive a kidney transplant

[1] Introduction, Overview:

- *Welcome, introduction of the research project and the researcher*
- *Establish a 'stop sign' for research participant to use if needing to pause the interview*

[2] Discussion of…:

Note for Interviewer: Subjects covered in this topic guide are to be explored openly – not leading. Appropriate prompts will be used – e.g. can you tell me more about that? How did that make you feel?

1. What is day-to-day living with ESKD like? (exploring different aspects of daily life – family life, school/college, employment, any treatment, limitations, participation in activities etc)
2. Which aspects of daily-living really matter to a family living with ESKD?
3. What are their experiences and beliefs around preparing for/receiving a kidney transplant?

[3] Conclusion:

1. *Summarise what has been discussed, mention the positive details, thank the young person / parent / carer*
2. "How did you find talking about living with chronic kidney disease?"
3. "Is there anything important to YOU that we have not talked about?"
4. Handle any concerns they have, signpost to any local services that could support their concerns.

For peer review only - http://bmjopen.bmj.com/site/about/guidelines.xhtml

An investigation of the modifiable psychosocial variables influencing access to and outcomes after kidney transplantation in children

Interview Topic Guide for Researcher – with the Young Person

Research Goals of the Interview:

4. Explore how the participant feels their life is now (with End Stage Kidney Disease [ESKD])
5. Explore what good quality life looks like to the participant – what matters most?
6. Explore what the participant believes is delaying or enabling the young person to receive a kidney transplant

[1] Introduction, Overview:

- *Welcome, introduction of the research project and the researcher*
- *Establish a 'stop sign' for research participant to use if needing to pause the interview*

[2] Discussion of…:

Note for Interviewer: Subjects covered in this topic guide are to be explored openly – not leading. Appropriate prompts will be used – e.g. can you tell me more about that? How did that make you feel?

4. What is day-to-day living with ESKD like? (exploring different aspects of daily life – family life, school/college, any treatment, limitations, participation in activities etc)
5. Which aspects of daily-living really matter to a family living with ESKD?
6. What are their experiences and beliefs around preparing for/receiving a kidney transplant?

[3] Conclusion:

5. *Summarise what has been discussed, mention the positive details, thank the young person / parent / carer*
6. "How did you find talking about living with chronic kidney disease?"
7. "Is there anything important to YOU that we have not talked about?"
8. Handle any concerns they have, signpost to any local services that could support their concerns.

For peer review only - http://bmjopen.bmj.com/site/about/guidelines.xhtml

An investigation of the modifiable psychosocial variables influencing access to and outcomes after kidney transplantation in children

Interview Topic Guide for Researcher – with NHS professional

Research Goals of the Interview:

7. Explore what the participant thinks matters most to their patients and families in terms of living a good quality life
8. Explore what the participant believes is delaying or enabling families to receive a kidney transplant from a mental health and social factors perspective

[1] Introduction, Overview:

- *Welcome, introduction of the research project and the researcher*
- *Establish a 'stop sign' for research participant to use if needing to pause the interview*

[2] Discussion of…:

Note for Interviewer: Subjects covered in this topic guide are to be explored openly – not leading. Appropriate prompts will be used – e.g. can you tell me more about that? Why do you think that is? How does that make you feel as a professional caring for these families?

7. From their experience of caring for families with ESKD, which aspects of daily-living really matter to these families?
8. What are their experiences of deciding whether a family is ready to prepare for kidney transplantation?
9. What are their experiences of families that do well and not so well after kidney transplantation?

[3] Conclusion:

9. *Summarise what has been discussed, mention the positive details, thank the NHS professional*
10. "How did you find the interview experience?"
11. "Is there anything important to YOU that we have not talked about?"
12. Handle any concerns they have, signpost to any local services that could support their concerns.

For peer review only - http://bmjopen.bmj.com/site/about/guidelines.xhtml

Supplementary Material 2

Demographic Background Survey

1. What is your child's ethnic group?
- a. White
- b. Black / Black-British
- c. Asian or Asian-British
- d. Mixed ethnicity
- e. Other ethnicity

2. How many main caregivers (e.g. parents) are in your family?
- a. One
- b. Two – living in same household
- c. Two – living in separate households
- d. Other _____________

3. What do you identify yourself as in relation to your child?
- a. Mother
- b. Father
- c. Aunt
- d. Uncle
- e. Grand mother
- f. Grand father
- g. Foster-carer
- h. Other _____________

4. What does the other main caregiver identify themselves as in relation to your child? (if applicable)
- a. Mother
- b. Father
- c. Aunt
- d. Uncle
- e. Grand mother
- f. Grand father
- g. Foster-carer
- h. Other _____________
- i. Not applicable

5. What is the highest level of education you have completed?
- a. Primary school
- b. Secondary school up to 16 years
- c. Higher or secondary or further education (e.g. A-levels, IB, BTEC)
- d. College or University
- **e.** Post-graduate degree

6. What is the highest level of education, which the other main caregiver has completed? (If applicable)
- a. Primary school
- b. Secondary school up to 16 years
- c. Higher or secondary or further education (e.g. A-levels, IB, BTEC)
- d. College or University
- e. Post-graduate degree
- f. Not applicable

7. What is your current employment status?
- **a.** Employed – full time
- **b.** Employed – Part time

For peer review only - http://bmjopen.bmj.com/site/about/guidelines.xhtml

c. Seeking employment
d. Home maker
e. Retired
f. Studying
g. Not seeking employment due to health reasons
h. Not seeking employment due to being full-time carer
i. Not seeking employment due to other reasons ________

8. What is the other main caregiver's current employment status? (If applicable)

a. Employed – full time
b. Employed – Part time
c. Seeking employment
d. Home maker
e. Retired
f. Studying
g. Not seeking employment due to health reasons
h. Not seeking employment due to being full-time carer
i. Not seeking employment due to other reasons __________
j. Not applicable

9. **Is there a recent family history of immigration into the United Kingdom?**

a. Yes, during my child or their sibling's lifetime (first generation)
b. Yes, during their parent's lifetime (second generation)
c. No recent history.

10. Have you had to move homes after your child was diagnosed with Chronic Kidney Disease?

a. Yes, home was inadequate for a child with Chronic Kidney Disease (e.g. unable to store dialysis equipment)
b. Yes, home was too far from child's hospital where they received care for their kidneys
c. Yes, but only temporarily (e.g. friend or relative's home or other temporary housing) because ____________
d. Yes, for other reasons _________________
e. Not applicable to our family

11. What is your family's total estimated income in the last year? (before tax, from all sources including salaries and benefits) Chronic Kidney Disease can have a knock-on effect on a family's circumstances, which is why we would like to understand this better by asking this information.

a. Up to £10,000
b. £10,001 to £20,000
c. £20,001 to £30,000
d. £30,001 to £50,000
e. £50,001 to £70,000
f. £70,001 to £150,000
g. Over £150,000
h. Prefer not to say

12. Next, we would like to ask you questions about your child's medications as this may have an effect on their quality of life.
Please select all of the kidney medicines your child is taking at the moment from this menu

- Aciclovir
- Azathioprine
- Calcium Acetate
- Calcium Carbonate
- Captopril
- Cefaclor
- Cefixim
- Ciclosporin (Sandimunn)
- Co-trimoxazole

For peer review only - http://bmjopen.bmj.com/site/about/guidelines.xhtml

- Darbepoetin
- Enalapril
- Epoetin
- Everolimus (Certican)
- Ganciclovir
- Growth Hormone
- Irbesartan
- Iron (Ferrous fumarate, Ferrous sulphate)
- Lactulose
- Losartan
- Movicol
- Mycophenolate Mofetil (MMF) (Cellcept, Myfenax)
- Nitrofurantoin
- Potassium Chloride
- Potassium Citrate
- Prednisolone
- Senna
- Sevelamer
- Sirolimus (Rapamune)
- Sodium Bicarbonate
- Sodium Chloride
- Tacrolimus (Advagraf, Prograf, Modigraf)
- Trimethoprim
- Valaciclovir
- Valganciclovir
- Vitamin D (Alfacalcidol)
- Vitamin D (Colecalciferol)
- Other(s)________ ________________

13. Please select how often your child takes these medicines (select all that apply):

a. Longer than every month
b. Every month
c. Every other week
d. Every week
e. Three times a week
f. Every other day
g. Once a day
h. Twice a day
i. Three times a day
j. Four times a day
k. More than four times a day
l. Other regime ___________

14. Please select how these medicines are given to your child (select all that apply)

a. An injection directly into their vein (via cannula or other access to their veins)
b. Other type of injection (e.g. into skin or muscle)
c. Tablets (by mouth)
d. Tablets (by gastrostomy tube)
e. Liquids (by mouth)
f. Liquids (by gastrostomy tube)
g. Eye drops
h. Creams / Lotions
i. Inhaler
j. Other type of medicine _____________

For peer review only - http://bmjopen.bmj.com/site/about/guidelines.xhtml

15. **Some families may have had their journey towards a kidney transplant affected by the COVID-19 pandemic. Please select which situation applies to your child and family due to the COVID-19 pandemic in 2020-2021:**
    a. Our family was not in the middle of preparing for a kidney transplant during 2020-2021. Therefore COVID-19 did not impact us.
    b. Our family already started or were starting to prepare for a kidney transplant in 2020-2021. The COVID-19 pandemic did not change our preparation plans.
    c. Our family delayed preparations for a kidney transplant from a living donor due to the COVID-19 pandemic.
    d. Our family came off the waiting list for a kidney transplant from a deceased donor due to the COVID-19 pandemic
    e. Our family delayed investigations that were part of preparing for a kidney transplant overall because of the COVID-19 pandemic
    f. Our family's transplant journey was affected by the COVID-19 pandemic is other ways ___________________________

For peer review only

How to investigate the modifiable psychosocial variables influencing access to and outcomes after kidney transplantation in children and young people

Journal:	*BMJ Open*
Manuscript ID	bmjopen-2023-078150.R1
Article Type:	Protocol
Date Submitted by the Author:	07-Apr-2024
Complete List of Authors:	Kim, Ji Soo; Great Ormond Street Hospital for Children NHS Foundation Trust, Paediatric Nephrology; NIHR Great Ormond Street Hospital Biomedical Research Centre<br>Wray, Jo; Great Ormond Street Hospital for Children NHS Foundation Trust, Centre for Outcomes and Experience Research in Children's Health, Illness and Disability<br>Ridout, Deborah; UCL, Population, Policy and Practice, UCL Great Ormond Street Institute of Child Health<br>Plumb, Lucy ; UK Renal Registry; Bristol Medical School, Population Health Sciences<br>Nitsch, Dorothea; London School of Hygiene & Tropical Medicine; UK Renal Registry<br>Robb, Matthew; NHS Blood and Transplant, Statistics and Clinical Studies<br>Paediatric Nephrology (BAPN), British Association for; British Association for Paediatric Nephrology<br>Marks, Dr. Stephen; Great Ormond Street Hospital For Children NHS Trust, Paediatric Nephrology; NIHR Great Ormond Street Hospital Biomedical Research Centre
**Primary Subject Heading**:	Paediatrics
Secondary Subject Heading:	Renal medicine, Qualitative research, Mental health
Keywords:	Renal transplantation < NEPHROLOGY, Paediatric transplant surgery < PAEDIATRIC SURGERY, Paediatric nephrology < NEPHROLOGY, Health Services Accessibility, Quality of Life, Social Support

How to investigate the modifiable psychosocial variables influencing access to and outcomes after kidney transplantation in children and young people

Ji Soo Kim[1,2], Jo Wray[3], Deborah Ridout[4], Lucy Plumb[5,6], Dorothea Nitsch[6,7], Matthew Robb[8], British Association for Paediatric Nephrology, Stephen D Marks[1,2]

1. Department of Paediatric Nephrology, Great Ormond Street Hospital for Children NHS Foundation Trust, Great Ormond Street London, UK
2. NIHR Great Ormond Street Hospital Biomedical Research Centre, University College London Great Ormond Street Institute of Child Health, London, UK
3. Centre for Outcomes and Experience Research in Children's Health, Illness and Disability, Great Ormond Street Hospital for Children NHS Foundation Trust, London, UK
4. Population, Policy and Practice Department, University College London Great Ormond Street Institute of Child Health, London, UK
5. Population Health Sciences, University of Bristol Medical School, Bristol, UK.
6. UK Renal Registry, Bristol, UK
7. Department of Non-communicable disease epidemiology, London School of Hygiene & Tropical Medicine
8. Statistics and Clinical Studies team at NHS Blood and Transplant, Bristol, UK

Address for Correspondence:
Professor Stephen D Marks, Dr Ji Soo Kim
Department of Paediatric Nephrology
Great Ormond Street Hospital for Children NHS Foundation Trust
London
WC1N 3JH
stephen.marks@gosh.nhs.uk jisoo.kim@nhs.net

Word Count: 3900/4000 words (excluding title page, abstract, tables, author's contribution, acknowledgments & references)
Abstract: 300/300 words

For peer review only - http://bmjopen.bmj.com/site/about/guidelines.xhtml

Abbreviations

CKD-5 – Stage V chronic kidney disease

KRT – kidney replacement therapy

MDT – multi-disciplinary team

QoL – Quality of Life

Keywords

Renal transplantation

Paediatric transplant surgery

Paediatric nephrology

Health Services Accessibility

Quality of Life

Social Support

For peer review only

Abstract

Introduction

Kidney transplantation is the preferred therapy for children with Stage 5 chronic kidney disease (CKD-5). However, there is wide variation in access to kidney transplantation across the UK for

For peer review only - http://bmjopen.bmj.com/site/about/guidelines.xhtml

children. This study aims to explore the psychosocial factors that influence access to and outcomes after kidney transplantation in children in the UK using a mixed methods prospective longitudinal design.

Methods

Qualitative data will be collected through semi-structured interviews with children affected by CKD-5, their carers and paediatric renal multi-disciplinary team. These interviews will inform the choice of existing validated questionnaires for the second phase of the study, during which selected questionnaires will be distributed to a larger national cohort of children with pre-transplant CKD-5 and their carers. Follow-up questionnaires will be sent at protocolised time points regardless of whether they receive a kidney transplant or not. Co-existing health data from hospital, UK renal registry and NHS Blood and Transplant registry records will be mapped to each questionnaire time point. An integrative analysis of the mixed qualitative and quantitative data will define psychosocial aspects of care for potential intervention to improve transplant access.

Analysis

Qualitative data will be analysed using thematic analysis. Quantitative data will be analysed using appropriate statistical methods to understand how these factors influence access to transplantation, as well as the distribution of psychosocial factors pre- and post-transplantation.

Ethics and Dissemination

This study protocol has been reviewed by the National Institute for Health Research Academy and approved by the Wales Research Ethics Committee 4 (IRAS number 270493/ref: 20/WA/0285) and the Scotland A Research Ethics Committee (ref: 21/SS/0038). Results from this study will be disseminated across media platforms accessed by affected families, presented at conferences, and published in peer-reviewed journals.

Registration Details

For peer review only - http://bmjopen.bmj.com/site/about/guidelines.xhtml

This work is supported by the National Institute for Health Research Academy, grant number NIHR300727.

For peer review only

For peer review only - http://bmjopen.bmj.com/site/about/guidelines.xhtml

Article Summary:

Strengths and limitations of this study

1. Prospective, longitudinal study design allows for a more detailed understanding of delays in transplantation associated with different psychosocial factors.
2. Combination of qualitative and quantitative data enable in-depth exploration of how psychosocial factors influence access to transplantation and outcomes thereafter.
3. Involvement of the majority of UK paediatric nephrology units ensuring representation of paediatric CKD-5 population
4. Limited follow-up timeline will capture only short to medium-term rather than long-term outcomes.
5. Utilisation of interpreters in qualitative interviews increases involvement of non-English speaking families, however the lack of validated translations of questionnaires may limit involvement of non-English speaking families in capturing quantitative data.

For peer review only

For peer review only - http://bmjopen.bmj.com/site/about/guidelines.xhtml

Introduction

Around 1,000 children (age 0-17 years) with Stage 5 chronic kidney disease (CKD-5) in the United Kingdom receive Kidney Replacement Therapy (KRT) in the form of either peritoneal dialysis, haemodialysis or kidney transplantation[1, 2]. Kidney transplantation is the gold standard therapy for reducing mortality and improving outcomes for children[3]. Minimising time on dialysis in favour of transplantation has been shown to reduce CKD-5 related complications and morbidity [4]. Compared with dialysis, transplantation is also presumed to improve patients' health-related quality of life (HR-QoL)[5]. Furthermore in the UK, for every year that the patient's kidney transplant functions, transplantation is three times more cost-effective than dialysis for the NHS[6].

However, not every child with CKD-5 can access kidney transplantation. There are approximately 193 to 217 prevalent children on dialysis each year[7, 8]. Annually only 130-160 paediatric kidney transplants are performed in the UK[9]. There appears to be variation in transplantation access, practice, and outcomes between UK paediatric nephrology units[1, 2, 9]. A cross-sectional survey conducted by the British Association for Paediatric Nephrology examined reasons for paediatric kidney transplantation delay in the UK. The survey showed that psychosocial factors make up 19% of the barriers, although specific factors were not identified in this study[10]. Compared with paediatrics, considerable research has been done in adult patients, regarding psychosocial factors implicated in transplant access. Formalised pre- and post-transplant psychosocial assessments have been widely researched for adult solid organ transplant recipients[11-14]. The UK-wide study 'Access to Transplantation and Transplant Outcome Measures' specifically investigated psychosocial barriers in adult kidney transplant recipients[15]. Researchers found inequities in transplant access, in spite of a universal healthcare system, based on socioeconomic status, education level, health literacy and racial background[16, 17]. In terms of outcomes, they found no difference in post-transplant HR-QoL between living and deceased donor recipients and that recipient expectations influenced post-transplant recovery[15-19]. However, for UK children, it is less clear what these 'psychosocial factors' are and how they influence unit-specific decisions, access to kidney transplantation and outcomes [10, 20, 21]. We acknowledge the recent progress

For peer review only - http://bmjopen.bmj.com/site/about/guidelines.xhtml

being made in exploring these psychosocial factors in some countries[22-24]. However, psychosocial studies for children with CKD-5 are still limited by retrospective or cross-sectional design or by single-centre or small study cohorts. There are no UK studies that prospectively explore how these factors impact paediatric kidney transplantation access over time.

Aims and Objectives:

This study aims to prospectively evaluate the psychosocial factors that are actual or perceived barriers to paediatric kidney transplantation which may be associated with poor transplant outcome. We anticipate these psychosocial factors to be broad at an individual, family and societal level and that they will encompass mental health and social determinants of health as defined by Marmot: 'the conditions in which people are born, grow, live, work and age'[25]– we hypothesise that psychosocial factors implicated in transplant access can be quantified using formal, validated measures, and that these factors may influence outcomes in the short-term period following transplantation.

This will be achieved through the following research objectives:

1. Describe the psychosocial factors perceived by clinicians as barriers to kidney transplantation.
2. Describe current interventions (if any) implemented to address these psychosocial factors.
3. Explore the experiences and beliefs of children and their families of any psychosocial challenges or facilitators in accessing a kidney transplant
4. Quantify the identified psychosocial factors implicated in CKD management
5. Measure the prevalence of identified psychosocial factors (positive and negative) in the national cohort of patients being pre-emptively worked up for transplant, on dialysis or listed for transplant.
6. Examine these psychosocial factors regarding their association with time to transplant and their changes following transplantation

For peer review only - http://bmjopen.bmj.com/site/about/guidelines.xhtml

7. Synthesise findings from each phase to inform recommendations about which psychosocial factors are potential barriers or facilitators to accessing kidney transplantation.

Findings synthesised from this study will inform development of a complex intervention to improve uptake of kidney transplantation in children with CKD-5 who would benefit most from one.

Methods and Analysis

Study Design Overview:

This prospective study has three phases, commencing with a sequential exploratory mixed-methods design, followed by a sequential explanatory mixed-methods design (Figure 1)[26]. This approach was chosen to first gain new insights by becoming familiar with the range of potential psychosocial factors and then longitudinally observing the influence of these factors on kidney transplantation access and outcomes for children[27]. Phase one will consist of exploratory interviews with purposively selected participants. These interviews will inform which questionnaires will be distributed at baseline and follow-up to the wider cohort of children with CKD-5 and their carer(s) in phase two stage A. Using the interviews to inform questionnaire selection will ensure questionnaires are relevant to, and resonate with, families. Participant families with outlier findings from phase two stage A will then be invited for explanatory interviews in phase two stage B. Finally, in phase three, the qualitative and quantitative data from the previous phases will be analysed together to develop an integrated understanding of psychosocial factors that influence access to kidney transplantation. All elements of the study design were co-developed and approved by our Research Partner Family and study steering group. Our Research Partner Family (a young person and parent dyad) and steering group members have lived experience of CKD, dialysis and kidney transplantation in childhood either as a patient or carer. The Great Ormond Street Hospital Young

For peer review only - http://bmjopen.bmj.com/site/about/guidelines.xhtml

Persons Advisory Group have also approved the research question and study design. The Young Persons Advisory Group consists of children affiliated with Great Ormond Street Hospital for Children NHS Foundation Trust as a patient or family member of a patient and therefore who have the lived experience needed to advise researchers designing studies involving children.

Population:

This study aims to include children, carer and staff participants from all 13 UK paediatric nephrology units, of which 10 units offer kidney transplantation surgery on site and 3 units which do not offer transplantation surgery at their own site but offer shared transplant care with nearby units.

In phase one and phase two stage A, children (aged 0 – 17 years inclusive) with CKD-5 on chronic dialysis, being worked up for pre-emptive kidney transplantation or on the waiting list for deceased donation (active or suspended) or awaiting living donation, will be invited to participate with their carer(s). Where appropriate, children aged 5 years and above will be invited to directly participate. For children aged under 5 years or those who are unable to assent or consent, their carers will be consulted and offered an opportunity to participate through proxy measures. Members of the multi-disciplinary team (MDT) involved in pre-transplantation work-up at their local unit (e.g., paediatric nephrologists, transplant surgeons, nurse specialists, social workers, family therapists, play therapists and members of the psychosocial team etc.) will be invited for interviews in Phase One only. Patients or carer(s) who are unable to give informed consent or patients who have been deemed too unwell to participate or had a recent acute hospital admission in the last 14 days will not be approached to participate in the study.

Phase One Interviews:

A purposive sampling matrix will be used to maximise the diversity of views. Criteria for sampling will include age, sex, and ethnicity of children, modality of KRT, whether the kidney unit offers

For peer review only - http://bmjopen.bmj.com/site/about/guidelines.xhtml

transplantation surgery or not and role in the MDT. Completed interviews will be analysed in parallel with ongoing recruitment. Participant recruitment will stop when no new themes are generated from the data.

Analysis of these interviews will contribute towards selecting questionnaires for use in phase two stage A.

<u>Phase Two Stage A Questionnaires:</u>

In this phase, we will aim to recruit every child with CKD-5 who meets the inclusion criteria at pre-transplant baseline with an anticipated recruitment rate of 60-70%.

At the time of study design, the predicted annual numbers across all 13 UK paediatric nephrology units as per UK Renal Registry (UKRR) reports were as follows[1, 2]:

- Number of dialysis patients on the transplant waiting-list in a year; n=70
- Number of patients starting dialysis in a year; n=125
- Number of patients not on dialysis but pre-emptively on the transplant waiting list; n=50
- Transplant rate; 130 to 160 patients per year

Based on these numbers, the predicted maximum number of eligible participants in a 1-year-period would be 245. We chose a 2-year-period for our sample size calculation, which would be a population size of 490, to include children who experience delays and receive their transplant after 1 year (See Figure 2). We plan to recruit 180 patients and after allowing for a 20% drop out, we expect to include 141 families. Assuming a prevalence of pertinent psychosocial factors in the national cohort of patients being pre-emptively worked up for transplant, on dialysis or listed for transplant of 15%, then with 141 families we will be able to estimate this with 5% precision. At the time of sample size calculation, there were no paediatric studies specifically measuring this nor studies encompassing all psychosocial factors. Therefore, we used the assumed prevalence of 15% based on a study measuring psychological distress in potential adult transplant candidates[28].

For peer review only - http://bmjopen.bmj.com/site/about/guidelines.xhtml

We expect some overlap in the cohort between Phase One and Phase Two Stage A and appreciate some families may develop research fatigue. Families who already took part in Phase One will be asked whether they would like to also take part in Phase Two Stage A or prefer to opt out.

Phase Two Stage B:

Results from phase two stage A will be reviewed for negative and positive cases. Families who have outlier findings, in terms of their answers to the validated questionnaires, will be invited to interview in phase two stage B, with the aim of reviewing and refining themes to develop additional theoretical explanation to the influence of psychosocial factors on transplant access and outcomes. Exact parameters to define outliers and sample size will be dependent on findings from phase two stage A.

Outcomes measured:

Phase One:

Using a Topic Guide (Supplementary Material 1), qualitative data will be collected in phase one to address research objectives 1-4 through semi-structured, in-depth interviews undertaken by the investigator (JSK). This format was chosen over focus groups to enable participation of younger or less verbal children and avoid further inconveniencing families through multiple research appointments from having separate interviews for young children and focus groups for carers. The family interview format will be based on the childs' decision to either interview with or separately from their carers, depending on which setting they find more comfortable. The research goals for family interviews will include exploring what families feel their life is like now living with CKD-5, what good HR-QoL looks like and what they believe delays or enables how soon they receive a kidney transplant. Similarly, research goals for MDT member interviews will include exploring what the professionals think matters most to families whose child has CKD-5 when it comes to a good HR-QoL and what they, as professionals, believe impacts how soon children access a kidney transplant in terms of psychosocial factors.

For peer review only - http://bmjopen.bmj.com/site/about/guidelines.xhtml

The Topic Guide has been developed from the investigator's systematic literature review on the subject matter and in consultation with the Young Persons Advisory Group and the Research Partner Family[29]. To minimise any inconvenience for joining the study, all participants will be interviewed using their preferred modality (telephone, video-call or face-to-face consultations) in keeping with COVID-19 safety recommendations[30]. Interview time with young children will be kept to less than half an hour to minimise interview fatigue. All interviews will be audio or video recorded depending on participant preference.

The acceptability of existing validated age-appropriate questionnaires that measure outcomes relevant to the preliminary themes, will be discussed with the steering group. Potential validated questionnaires that capture the preliminary themes will be identified from the systematic literature review and a wider search of the literature. These questionnaires will be checked in terms of their psychometric properties such as internal consistency (Cronbach alpha of at least 0.7) and test re-test reliability and aspects such as availability of the measures, respondent type (parent, child, or other respondent) and age range for which the questionnaire has been validated, to enable the most appropriate questionnaire to be chosen to measure each theme. The selected questionnaires will then be discussed with the steering group, considering the language of the questionnaire, acceptability, and level of burden for the participant. If the list of questionnaires is too onerous for the participating family, a consensus will be reached with the steering group on which preliminary themes and therefore which questionnaires should be prioritised. Once the final list of questionnaires is agreed on, these will be submitted to the Health Research Authority for final approval.

For peer review only

<u>Phase Two Stage A:</u>

In phase two stage A, research objectives 5-6 will be addressed by measuring the following primary outcome variables: HR-QoL, psychosocial functioning and time taken to receive a kidney transplant since date confirming CKD-5. To understand changes in psychosocial factors over time, their associations with health burden or short-term allograft deterioration must be accounted for,

For peer review only - http://bmjopen.bmj.com/site/about/guidelines.xhtml

Therefore, we will measure the following secondary outcome: the child's estimated glomerular filtration rate (eGFR) over time. The eGFR will be calculated with their height and serum creatinine using the CkiD U25 formula [31, 32].

Questionnaires measuring HR-QoL and psychosocial functioning, selected from phase one, will be distributed to a larger, national cohort of children with CKD-5 and their carers at their pre-transplant baseline. Follow-up questionnaires will be sent post-transplant at 3-, 6- and 12-months later or 12-months after their first questionnaire if they still have not received a kidney transplant in that time frame. These follow-up time points were chosen to reflect the initial period of post-transplant adaptation, which are comparable with similar studies in adults and children at the time of protocol-writing[33-35]. For children who have not received a transplant, a 12-month interval was advised by our Steering Group to avoid distress triggered by frequent questionnaires reminding them of their non-transplanted state. As families are more likely to participate if they can choose which questionnaire modality is most suited to their lifestyle, participant families will be offered either paper or online questionnaires[36]. Health morbidity and co-existing disease data, including their underlying primary kidney diagnosis, as described in Table 1, will be mapped to each questionnaire time point.

Baseline of all participating CYP	CYP not transplanted yet at 12 months follow-up	CYP who are Post-transplant
• Ethnicity<br>• Primary renal diagnosis<br>• Other co-morbidity diagnoses<br>• Modality of KRT if applicable<br>• Date confirming CKD-5<br>• Date of starting RRT if applicable<br>• Date of previous kidney transplant reaching CKD-5 if applicable<br>• Height (cm)<br>• Weight (kg)<br>• Serum Creatinine (ummol/L)	• Modality of KRT if changed (if applicable)<br>• Date of starting new modality of RRT (if applicable)<br>• Height (cm)<br>• Weight (kg)<br>• Serum Creatinine (ummol/L)<br>• Number and type of medications*	<u>Only at 3 months post-transplant</u><br>• Type of transplant kidney (Living related / Living Unrelated / Deceased Donor (after cardiac or brain death)<br>• Donor & recipient mismatch<br>• ABO compatibility<br>• Cold ischaemic Time<br>• Need for plasma exchange / immunoadsorption / Rituximab / iv immunoglobulin<br><u>At all post-transplant intervals (3-, 6-, 12- and 24-months)</u><br>• Changes to co-morbidities if applicable<br>• Change of KRT modality if applicable<br>• Date of RRT modality change if applicable<br>• Height (cm)

For peer review only - http://bmjopen.bmj.com/site/about/guidelines.xhtml

• Number and type of medications<br>• Post code*		• Weight (kg)<br>• Serum Creatinine (ummol/L)<br>• Number and type of medications

Table 1: Co-existing Health Data to be collected and mapped against questionnaire time points

** Post code data will be converted to the relevant national multiple indices of deprivation (e.g. English Indices of Deprivation, Scottish Index of Multiple Deprivation)*

An additional questionnaire (see Supplementary Material 2) has been co-designed with the steering group to retrieve information on the participating family's demographic background. Data on medication burden will also be collected due to its' possible confounding effect HR-QoL as described in current literature[37].

Phase Two Stage B:

The Interview Topic Guide for Phase Two Stage B has been designed with flexibility, since the interview will depend on the nature of the outlier findings from Phase Two Stage A.

Data Analysis Plan:

Phase One:

To address research objectives 1-4, JSK will thematically analyse interview transcripts, following the approach of Braun and Clarke [38]. De-identified transcripts will be managed using Nvivo software[39]. For the purposes of qualitative rigour, JSK will maintain a reflexivity journal after interviews and throughout data analysis, to ensure potential researcher biases and insights are noted. Maintaining a reflexivity journal is a gold standard practice in qualitative research, which increases the credibility and deepens the understanding of the findings by describing the context

For peer review only - http://bmjopen.bmj.com/site/about/guidelines.xhtml

in which data were collected and analysed[40, 41]. A sample of transcripts will be examined by JW and discussed with JSK. Throughout all stages of data collection and analysis, findings will be shared and discussed by JSK, JW and SDM.

Phase Two Stage A:

Participant demographics will be described using descriptive statistics. It will also be used to address research objective 4 by describing the prevalence of psychosocial factors.

Research objective 6 will be addressed through the following:

First, descriptive statistics will again be utilised to describe how participants' psychosocial functioning and HR-QoL variables change before and after transplantation at each data collection time point.

Second, to describe the impact waiting to access a kidney transplant and receiving one has on psychosocial functioning and HR-QoL over time, repeated measure ANOVA will be undertaken.

Third, the association between clinical, demographic, and psychosocial factors with accessing a kidney transplant will be measured using logistic regression modelling and Kaplan-Meier statistics.

Fourth, the association of clinical, demographic, and psychosocial factors with failing transplant allograft in short-term follow-up between one- and two-years post-transplantation will be assessed. Where the outcome variable is the child's eGFR, linear regression modelling will be used. Where the outcome variable defines a failing post-transplant kidney as reaching an eGFR equivalent to stage IV CKD (eGFR 15-29 ml/min/1.73m^2), logistic regression modelling and Kaplan-Meier statistics will be utilised.

Separately, the level of agreement between child and carer responses will be assessed. Choice of statistical tests will depend on the type of data: for example, Cohen's Kappa for binary data, weighted Kappa for ordinal data or interclass correlation coefficients for continuous data.

For peer review only - http://bmjopen.bmj.com/site/about/guidelines.xhtml

Where relevant, all statistical analyses will be adjusted for (but not limited to) child's age, gender, ethnicity, socioeconomic status, and KRT status at baseline.

Phase Two Stage B:

Interview data will be analysed using Thematic Analysis underpinned by the same principles as in phase one.

Phase Three:

An integrative analysis of data from phases one and two will be undertaken to address research objective 7 and create a conceptual model of how psychosocial factors influence transplant access and outcomes. The quantitative and qualitative data will be reviewed together to understand where they converge, complement, or diverge from the other dataset or have findings that are novel. A final lay report will be co-written with the steering group to ensure it is meaningful, relevant, and accessible to families whose child have CKD.

Ethics

This study protocol has been peer reviewed by the National Institute for Health Research Academy and has been approved under IRAS number 270493 by the Wales Research Ethics Committee 4 (ref: 20/WA/0285) and the Scotland A Research Ethics Committee (ref: 21/SS/0038).

Patient and public involvement, engagement and participation is integral to ensuring that all aspects of study procedure and materials are ethically sound and acceptable to participant families and children of different ages. As aforementioned, the study design and all participant-facing materials were discussed with the Young Persons Advisory Group, the Research Partner Family and the steering group.

For peer review only - http://bmjopen.bmj.com/site/about/guidelines.xhtml

For peer review only

Coercive pressure of joining the study will be minimised. Research participants will not be paid for participation. Participant information sheets will indicate that there will be no added benefit or disruption to their medical care.

Children will be encouraged to be an active participant by obtaining Informed written consent from all participants aged ≥16 years old and written assent from participants aged 5 – 15 years old. Consent will be obtained for all research activities including interviews, questionnaires and retrieving health information from national databases and hospital records.

To ensure no participant is unfairly excluded in phase one interviews due to English not being their first language, interpreters will be present to support their participation. The same principle of minimising communication barriers will be applied for younger children or young people who may prefer communicating through other creative outputs such as drawing or Talking Mats. Ideally similar principles would be applied to phase two questionnaires, but the availability of translated and validated questionnaires is a limitation.

Some children may prefer their carers to be present to help express themselves more fully whereas some may prefer their carers to be absent to express themselves more freely. Therefore, all children will be given the option to be interviewed with or without their carer in the room.

Participants in the phase one interviews may become distressed as they reflect on their experiences due to the sensitive nature of some of the interview topics around their mental health or transplant delays. Therefore, the participant and JSK will agree on a 'stop signal' before commencing the interview for use should they feel uncomfortable. If the participant uses the 'stop signal', they will be offered a break. The participant and JSK will then discuss whether they would like to continue, reschedule or withdraw from the interview altogether. Once the interview recording stops, there will be an opportunity to discuss the participant's feelings and if appropriate, they will be signposted to their local support services.

Participant confidentiality will be upheld by fully adhering to the Data Protection Act. Participant identifiers will be handled with appropriate pseudonymisation and all data will be kept on GDPR-compliant encrypted devices or stored on hard drives with restricted access with the relevant

For peer review only - http://bmjopen.bmj.com/site/about/guidelines.xhtml

encryption and password protection. The only instance where confidentiality will be breached is if a participant discloses information that has direct implications for child or adult safeguarding. Potential participants will be made aware of this as part of informed consent. All interviews will be digitally recorded, transcribed verbatim and have identifiable data redacted. Audio files will be transcribed either by JSK or by a third-party interview transcription company (Take Note®). To minimise data handling breaches, all engagement with Take Note® will only be through their secure web platform. All video files will be transcribed only by JSK to remove third party involvement in the de-identification process. Once de-identified, only quotes that cannot lead to participant identification will be used in reports. Care will be taken in reporting findings to ensure individuals cannot be identified by their role, diagnosis, gender, age or geographic locality.

Dissemination

Given this project's relevance to both patient families and their clinicians, a targeted dissemination strategy will be adopted. Research participants will be updated about the findings through newsletters. Lay summaries approved by the steering group will be disseminated across charity websites accessed by children and families affected by CKD. To ensure that professionals who work with families affected by CKD are being reached, findings will be disseminated widely in relevant peer-reviewed journals and at national and international conferences. Finally, if appropriate, a dissemination strategy will be co-created between JSK and the steering group for other professionals (e.g., teachers) who may come into contact with vulnerable families with CKD who may need early referral for psychosocial intervention.

Conclusion

For peer review only - http://bmjopen.bmj.com/site/about/guidelines.xhtml

Psychosocial factors influencing access to and outcomes after paediatric kidney transplantation are poorly understood. As almost one fifth of eligible children are delayed from receiving kidney transplantation due to psychosocial factors, this is an important area of study[10]. The mixed-methods, prospective, and longitudinal design of this research project, has the potential to advance our understanding of how these factors truly influence access to transplantation in a way that a retrospective or cross-sectional study cannot. Findings from this project will be able to be utilised by UK paediatric nephrology units to optimise and standardise their psychosocial care for children being prepared for kidney transplantation. Finally, findings will also inform future research into developing interventions that better target aspects of psychosocial support for children and their families to improve their access to and short-term outcomes of kidney transplantation.

For peer review only

Author's contribution

- JSK wrote the study protocol and coordinated the entire manuscript.
- Deborah Ridout contributed towards sample size calculation and statistical methods of study protocol.
- LP contributed towards statistical analysis of protocol and advised regarding UK Renal Registry involvement.
- DN contributed towards statistical analysis of protocol and advised regarding UK Renal Registry involvement.
- MR contributed towards statistical analysis of protocol and advised regarding NHS Blood & Transplant involvement.
- BAPN contributed towards study protocol development in terms of multi-centre data collection feasibility.
- JW supervised JSK, study protocol development with regular input to initial drafts and final manuscript approval.
- SDM supervised JSK, study protocol development with regular input to initial drafts and final manuscript approval.

For peer review only

Acknowledgements

We would like to thank the Great Ormond Street Hospital Young Person's Advisory Group and members of our Research Partner Family Steering Group –Emma Beeden, Katy Beeden, Angela Watt and Heather Davis from KDARS (Kidney Disease and Renal Support) for kids, for their expertise, support and advice. Finally, we would also like to thank members of the British Association for Paediatric Nephrology, the National Kidney Federation and Kidney Care UK for their continued support of this study.

Funding Statement:

This work is supported by the National Institute for Health Research Academy, as a Doctoral Fellowship, grant number NIHR300727.

Competing Interests Statement:

Dr Ji Soo Kim is the National Institute for Health Research Fellowship grant recipient, which funds this study.

Dr Lucy Plumb reports grants from the National Institute for Health Research and Kidney Research UK. She is also the paediatric research lead for the UK Renal Registry.

Figure Legends

Figure 1: Flow diagram of sequential mixed method study design

Figure 2: Flow diagram illustrating predicted number of CYP who are eligible for transplantation and receive one across two years

For peer review only

References

1. Plumb L, Casula A, Pyart R, Evans KM, Inward C, Medcalf J, et al. The 21st UK Renal Registry Annual Report: A Summary of Analyses of Paediatric Data in 2017. Nephron. 2019:1-5.
2. Plumb L, Wong E, Casula A, Braddon F, Lewis M, Marks SD, et al. Chapter 4 Demography of the UK Paediatric Renal Replacement Therapy Population in 2016. Nephron. 2018;139 Suppl 1:105-16.
3. Wolfe RA, Ashby VB, Milford EL, Ojo AO, Ettenger RE, Agodoa LYC, et al. Comparison of Mortality in All Patients on Dialysis, Patients on Dialysis Awaiting Transplantation, and Recipients of a First Cadaveric Transplant. New England Journal of Medicine. 1999;341(23):1725-30.
4. Pruthi R, Casula A, Inward C, Roderick P, Sinha MD. Early Requirement for RRT in Children at Presentation in the United Kingdom: Association with Transplantation and Survival. Clin J Am Soc Nephrol. 2016;11(5):795-802.
5. Park KS, Hwang Yj Fau - Cho MH, Cho Mh Fau - Ko CW, Ko Cw Fau - Ha IS, Ha Is Fau - Kang HG, Kang Hg Fau - Cheong HI, et al. Quality of life in children with end-stage renal disease based on a PedsQL ESRD module. 2012(1432-198X (Electronic)).
6. Kerr M, Bray B, Medcalf J, O'Donoghue DJ, Matthews B. Estimating the financial cost of chronic kidney disease to the NHS in England. Nephrol Dial Transplant. 2012;27 Suppl 3:iii73-80.
7. UKRR. UK Renal Registry 21st Annual Report - data to 31/12/2017 Bristol, UK2019 [cited 2023. Available from: https://www.renalreg.org/publications-reports/.
8. UKRR. UK Renal Registry 24th Annual Report - data to 31/12/2020 Bristol, UK2022 [Available from: https://ukkidney.org/audit-research/annual-report.
9. Transplant OD. Annual Report on Kidney Transplantation Report for 2016/2017 NHS Blood and Transplant2017 [Available from: https://nhsbtdbe.blob.core.windows.net/umbraco-assets-corp/4607/kidney-annual-report-2016-17.pdf.
10. Kim JS, Marlais M, Balasubramanian R, Muorah M, Inward C, Smith GC, et al. UK national study of barriers to renal transplantation in children. Archives of disease in childhood. 2020.
11. Feurer ID, Moore DE, Speroff T, Liu H, Payne J, Harrison C, et al. Refining a health-related quality of life assessment strategy for solid organ transplant patients. Clin Transplant. 2004;18 Suppl 12:39-45.
12. Goetzmann L, Ruegg L, Stamm M, Ambuhl P, Boehler A, Halter J, et al. Psychosocial profiles after transplantation: a 24-month follow-up of heart, lung, liver, kidney and allogeneic bone-marrow patients. Transplantation. 2008;86(5):662-8.
13. Maldonado JR, Sher Y, Lolak S, Swendsen H, Skibola D, Neri E, et al. The Stanford Integrated Psychosocial Assessment for Transplantation: A Prospective Study of Medical and Psychosocial Outcomes. Psychosom Med. 2015;77(9):1018-30.
14. Shah VS, Ananth A, Sohal GK, Bertges-Yost W, Eshelman A, Parasuraman RK, et al. Quality of life and psychosocial factors in renal transplant recipients. Transplant Proc. 2006;38(5):1283-5.
15. Wu DA, Robb ML, Watson CJE, Forsythe JLR, Tomson CRV, Cairns J, et al. Barriers to living donor kidney transplantation in the United Kingdom: a national observational study. Nephrol Dial Transplant. 2017;32(5):890-900.
16. Pruthi R, Robb ML, Oniscu GC, Tomson C, Bradley A, Forsythe JL, et al. Inequity in Access to Transplantation in the United Kingdom. Clin J Am Soc Nephrol. 2020;15(6):830-42.
17. Taylor DM, Bradley JA, Bradley C, Draper H, Dudley C, Fogarty D, et al. Limited health literacy is associated with reduced access to kidney transplantation. Kidney Int. 2019;95(5):1244-52.

For peer review only - http://bmjopen.bmj.com/site/about/guidelines.xhtml

18. Wu DA, Robb ML, Forsythe JLR, Bradley C, Cairns J, Draper H, et al. Recipient Comorbidity and Survival Outcomes after Kidney Transplantation: a UK-wide Prospective Cohort Study. Transplantation. 2019.
19. Gibbons A, Bayfield J, Cinnirella M, Draper H, Johnson RJ, Oniscu GC, et al. Changes in quality of life (QoL) and other patient-reported outcome measures (PROMs) in living-donor and deceased-donor kidney transplant recipients and those awaiting transplantation in the UK ATTOM programme: a longitudinal cohort questionnaire survey with additional qualitative interviews. BMJ Open. 2021;11(4):e047263.
20. Fung E, Shaw RJ. Pediatric Transplant Rating Instrument - a scale for the pretransplant psychiatric evaluation of pediatric organ transplant recipients. Pediatr Transplant. 2008;12(1):57-66.
21. Killian MO, Schuman DL, Mayersohn GS, Triplett KN. Psychosocial predictors of medication non-adherence in pediatric organ transplantation: A systematic review. Pediatr Transplant. 2018;22(4):e13188.
22. Anand A, Malik TH, Dunson J, McDonald MF, Christmann CR, Galvan NTN, et al. Factors associated with long-term graft survival in pediatric kidney transplant recipients. Pediatr Transplant. 2021;25(4):e13999.
23. Freiberger D, Kimball B, Traum AZ, Berbert L, O'Melia L, Daly KP, et al. Equity factors in pediatric transplant listing: Initial findings from a single center review. Pediatr Transplant. 2023;27(2):e14467.
24. Rea KE, West KB, Dorste A, Christofferson ES, Lefkowitz D, Mudd E, et al. A systematic review of social determinants of health in pediatric organ transplant outcomes. Pediatr Transplant. 2023;27(1):e14418.
25. Marmot MA, T; Bell, J; Black, C; Broadfoot, P; Cumberlege, J; Diamond, I; Gilmore, I; Ham, C; Meacher, M; Mulgan, G. Fair Society, Healthy Lives, The Marmot Review: Institute of Health Equity; 2010 [Available from: https://www.instituteofhealthequity.org/resources-reports/fair-society-healthy-lives-the-marmot-review.
26. Creswell JW. A Concise Introduction to Mixed Methods Research. 1 ed. Thousand Oaks, United States: SAGE Publications Inc; 2014.
27. Reiter B. Theory and Methodology of Exploratory Social Science Research. International Journal of Science and Research Methodology 2017;5(4):129-50.
28. Kuntz KK, Bonfiglio DB. Psychological distress in patients presenting for initial renal transplant evaluation. J Clin Psychol Med Settings. 2011;18(3):307-11.
29. Kim J, Chesters H, Wray J, Marks SD. What are the psychosocial factors that influence access to kidney transplantation (and their outcomes) for children and young people? A Systematic Literature Review Protocol University of York: PROSPERO; 2020 [cited 2021. Available from: https://www.crd.york.ac.uk/PROSPEROFILES/210506_PROTOCOL_20210413.pdf.
30. BAPN. COVID-19 Updated Shielding Guidance for Children and Young People with Kidney Disease UK: Renal Association; 2020 [cited 2020. Available from: https://renal.org/wp-content/uploads/2020/06/Shielding-guidance-25.06.2020.pdf.
31. Ng DK, Pierce CB. Kidney Disease Progression in Children and Young Adults With Pediatric CKD: Epidemiologic Perspectives and Clinical Applications. Semin Nephrol. 2021;41(5):405-15.
32. Pierce CB, Muñoz A, Ng DK, Warady BA, Furth SL, Schwartz GJ. Age- and sex-dependent clinical equations to estimate glomerular filtration rates in children and young adults with chronic kidney disease. Kidney Int. 2021;99(4):948-56.
33. Kugler C, Gottlieb J, Warnecke G, Schwarz A, Weissenborn K, Barg-Hock H, et al. Health-related quality of life after solid organ transplantation: a prospective, multiorgan cohort study. Transplantation. 2013;96(3):316-23.
34. Wightman A, Bradford MC, Smith J. Health-related quality of life changes following renal transplantation in children. Pediatr Transplant. 2019;23(2):e13333.
35. Wyld M, Morton RL, Hayen A, Howard K, Webster AC. A systematic review and meta-analysis of utility-based quality of life in chronic kidney disease treatments. PLoS Med. 2012;9(9):e1001307.

For peer review only - http://bmjopen.bmj.com/site/about/guidelines.xhtml

36. Pulham RA, Wray J, Feinstein Y, Brown K, Pierce C, Nadel S, et al. Feasibility and Acceptability of Methods to Collect Follow-Up Information From Parents 12 Months After Their Child's Emergency Admission to Pediatric Intensive Care. Pediatr Crit Care Med. 2019;20(4):e199-e207.
37. Díaz-González de Ferris ME, Pierce CB, Gipson DS, Furth SL, Warady BA, Hooper SR. Health-related quality of life in children with chronic kidney disease is affected by the number of medications. Pediatr Nephrol. 2021;36(5):1307-10.
38. Braun V, Clarke V. Using thematic analysis in psychology. Qualitative Research in Psychology. 2006;3(2):77-101.
39. Lumivero. NVivo Denver2020 [cited 2023 2023]. 2020:[Available from: https://lumivero.com/products/nvivo/.
40. Dodgson JE. Reflexivity in Qualitative Research. J Hum Lact. 2019;35(2):220-2.
41. Russell GM, Kelly NH. Research as Interacting Dialogic Processes: Implications for Reflexivity. Forum Qualitative Sozialforschung / Forum: Qualitative Social Research. 2002;3(3).

For peer review only

For peer review only - http://bmjopen.bmj.com/site/about/guidelines.xhtml

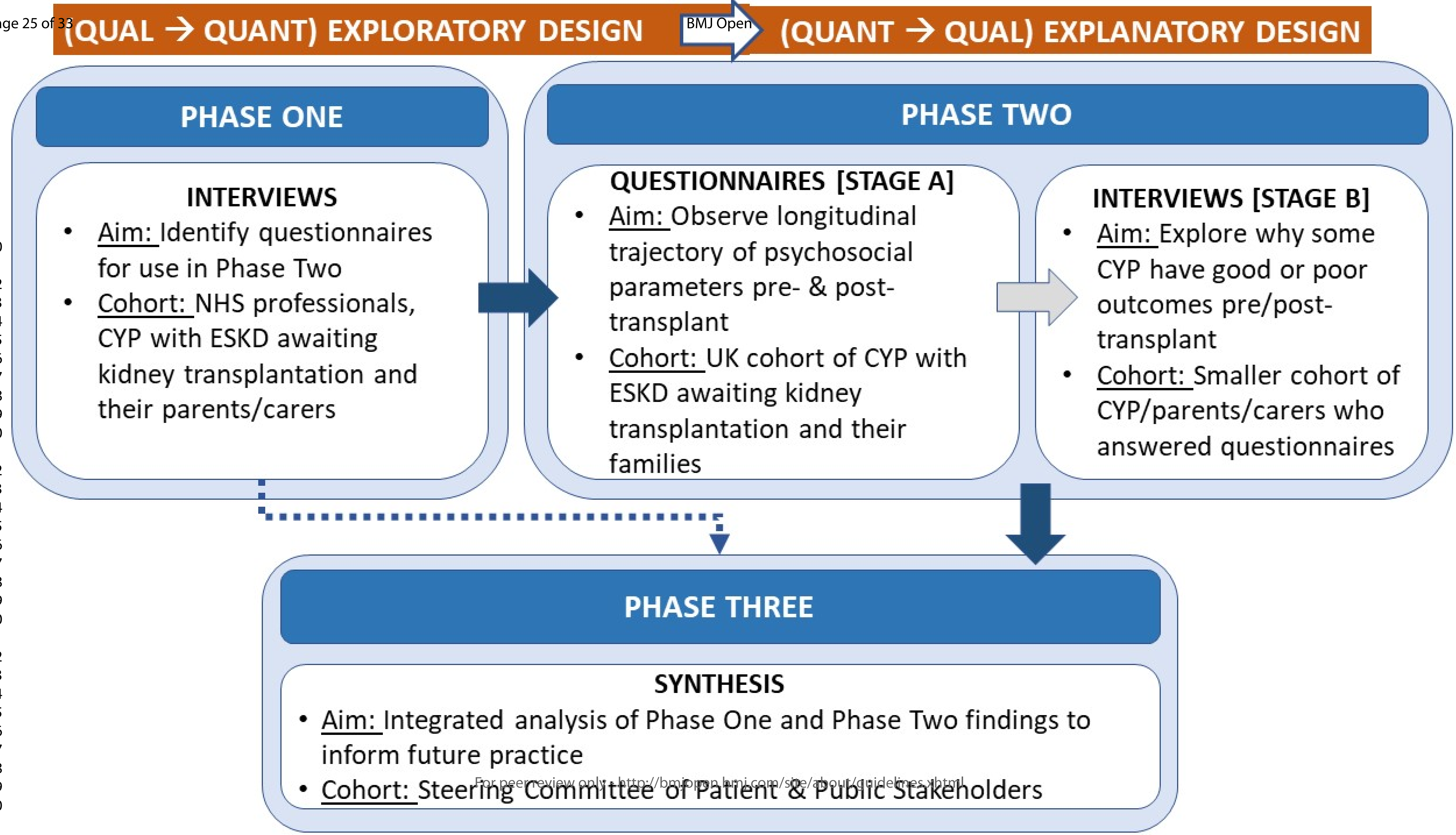
(QUAL → QUANT) EXPLORATORY DESIGN
(QUANT → QUAL) EXPLANATORY DESIGN
PHASE ONE
INTERVIEWS
• Aim: Identify questionnaires for use in Phase Two
• Cohort: NHS professionals, CYP with ESKD awaiting kidney transplantation and their parents/carers
PHASE TWO
QUESTIONNAIRES [STAGE A]
• Aim: Observe longitudinal trajectory of psychosocial parameters pre- & post-transplant
• Cohort: UK cohort of CYP with ESKD awaiting kidney transplantation and their families
INTERVIEWS [STAGE B]
• Aim: Explore why some CYP have good or poor outcomes pre/post-transplant
• Cohort: Smaller cohort of CYP/parents/carers who answered questionnaires
PHASE THREE
SYNTHESIS
• Aim: Integrated analysis of Phase One and Phase Two findings to inform future practice
• Cohort: Steering Committee of Patient & Public Stakeholders

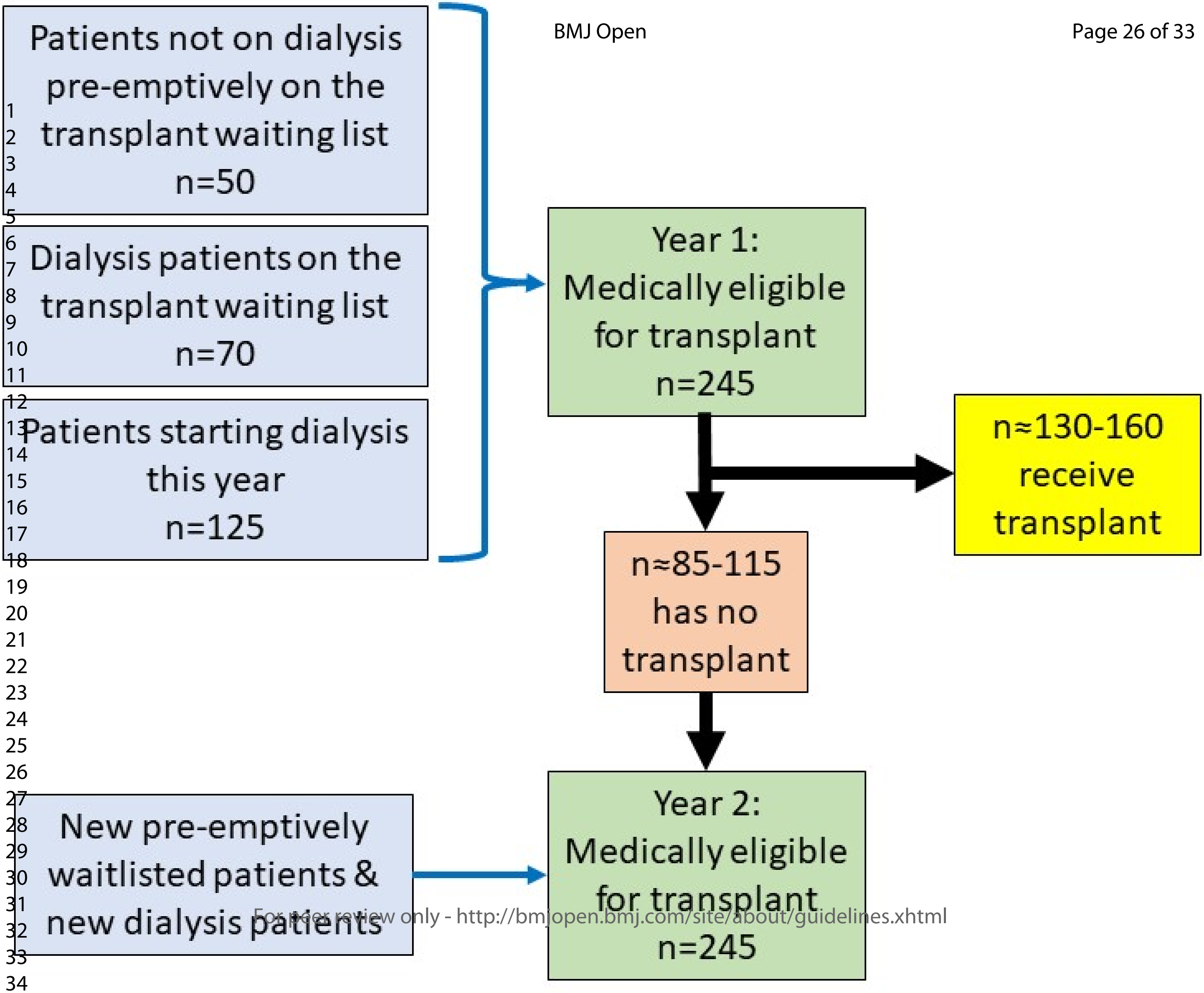

For peer review only - http://bmjopen.bmj.com/site/about/guidelines.xhtml

Supplementary Material 1

An investigation of the modifiable psychosocial variables influencing access to and outcomes after kidney transplantation in children

Interview Topic Guide for Researcher – with parent / guardian

Research Goals of the Interview:

1. Explore how the participant feels their life is now (with End Stage Kidney Disease [ESKD])
2. Explore what good quality life looks like to the participant – what matters most?
3. Explore what the participant believes is delaying or enabling their child to receive a kidney transplant

[1] Introduction, Overview:

- *Welcome, introduction of the research project and the researcher*
- *Establish a 'stop sign' for research participant to use if needing to pause the interview*

[2] Discussion of…:

Note for Interviewer: Subjects covered in this topic guide are to be explored openly – not leading. Appropriate prompts will be used – e.g. can you tell me more about that? How did that make you feel?

1. What is day-to-day living with ESKD like? (exploring different aspects of daily life – family life, school/college, employment, any treatment, limitations, participation in activities etc)
2. Which aspects of daily-living really matter to a family living with ESKD?
3. What are their experiences and beliefs around preparing for/receiving a kidney transplant?

[3] Conclusion:

1. *Summarise what has been discussed, mention the positive details, thank the young person / parent / carer*
2. "How did you find talking about living with chronic kidney disease?"
3. "Is there anything important to YOU that we have not talked about?"
4. Handle any concerns they have, signpost to any local services that could support their concerns.

For peer review only - http://bmjopen.bmj.com/site/about/guidelines.xhtml

An investigation of the modifiable psychosocial variables influencing access to and outcomes after kidney transplantation in children

Interview Topic Guide for Researcher – with the Young Person

Research Goals of the Interview:

4. Explore how the participant feels their life is now (with End Stage Kidney Disease [ESKD])
5. Explore what good quality life looks like to the participant – what matters most?
6. Explore what the participant believes is delaying or enabling the young person to receive a kidney transplant

[1] Introduction, Overview:

- *Welcome, introduction of the research project and the researcher*
- *Establish a 'stop sign' for research participant to use if needing to pause the interview*

[2] Discussion of…:

Note for Interviewer: Subjects covered in this topic guide are to be explored openly – not leading. Appropriate prompts will be used – e.g. can you tell me more about that? How did that make you feel?

4. What is day-to-day living with ESKD like? (exploring different aspects of daily life – family life, school/college, any treatment, limitations, participation in activities etc)
5. Which aspects of daily-living really matter to a family living with ESKD?
6. What are their experiences and beliefs around preparing for/receiving a kidney transplant?

[3] Conclusion:

5. *Summarise what has been discussed, mention the positive details, thank the young person / parent / carer*
6. "How did you find talking about living with chronic kidney disease?"
7. "Is there anything important to YOU that we have not talked about?"
8. Handle any concerns they have, signpost to any local services that could support their concerns.

For peer review only - http://bmjopen.bmj.com/site/about/guidelines.xhtml

An investigation of the modifiable psychosocial variables influencing access to and outcomes after kidney transplantation in children

Interview Topic Guide for Researcher – with NHS professional

Research Goals of the Interview:

7. Explore what the participant thinks matters most to their patients and families in terms of living a good quality life
8. Explore what the participant believes is delaying or enabling families to receive a kidney transplant from a mental health and social factors perspective

[1] Introduction, Overview:

- *Welcome, introduction of the research project and the researcher*
- *Establish a 'stop sign' for research participant to use if needing to pause the interview*

[2] Discussion of…:

Note for Interviewer: Subjects covered in this topic guide are to be explored openly – not leading. Appropriate prompts will be used – e.g. can you tell me more about that? Why do you think that is? How does that make you feel as a professional caring for these families?

7. From their experience of caring for families with ESKD, which aspects of daily-living really matter to these families?
8. What are their experiences of deciding whether a family is ready to prepare for kidney transplantation?
9. What are their experiences of families that do well and not so well after kidney transplantation?

[3] Conclusion:

9. *Summarise what has been discussed, mention the positive details, thank the NHS professional*
10. "How did you find the interview experience?"
11. "Is there anything important to YOU that we have not talked about?"
12. Handle any concerns they have, signpost to any local services that could support their concerns.

For peer review only

Supplementary Material 2

Demographic Background Survey

1. What is your child's ethnic group?

a. White
b. Black / Black-British
c. Asian or Asian-British
d. Mixed ethnicity
e. Other ethnicity

2. How many main caregivers (e.g. parents) are in your family?

a. One
b. Two – living in same household
c. Two – living in separate households
d. Other _____________

3. What do you identify yourself as in relation to your child?

a. Mother
b. Father
c. Aunt
d. Uncle
e. Grand mother
f. Grand father
g. Foster-carer
h. Other _____________

4. What does the other main caregiver identify themselves as in relation to your child? (if applicable)

a. Mother
b. Father
c. Aunt
d. Uncle
e. Grand mother
f. Grand father
g. Foster-carer
h. Other _____________
i. Not applicable

5. What is the highest level of education you have completed?

a. Primary school
b. Secondary school up to 16 years
c. Higher or secondary or further education (e.g. A-levels, IB, BTEC)
d. College or University
e. Post-graduate degree

6. What is the highest level of education, which the other main caregiver has completed? (If applicable)

a. Primary school
b. Secondary school up to 16 years
c. Higher or secondary or further education (e.g. A-levels, IB, BTEC)
d. College or University
e. Post-graduate degree
f. Not applicable

7. What is your current employment status?

a. Employed – full time
b. Employed – Part time

For peer review only

For peer review only - http://bmjopen.bmj.com/site/about/guidelines.xhtml

c. Seeking employment
d. Home maker
e. Retired
f. Studying
g. Not seeking employment due to health reasons
h. Not seeking employment due to being full-time carer
i. Not seeking employment due to other reasons ________

8. What is the other main caregiver's current employment status? (If applicable)

a. Employed – full time
b. Employed – Part time
c. Seeking employment
d. Home maker
e. Retired
f. Studying
g. Not seeking employment due to health reasons
h. Not seeking employment due to being full-time carer
i. Not seeking employment due to other reasons __________
j. Not applicable

9. **Is there a recent family history of immigration into the United Kingdom?**

a. Yes, during my child or their sibling's lifetime (first generation)
b. Yes, during their parent's lifetime (second generation)
c. No recent history.

10. Have you had to move homes after your child was diagnosed with Chronic Kidney Disease?

a. Yes, home was inadequate for a child with Chronic Kidney Disease (e.g. unable to store dialysis equipment)
b. Yes, home was too far from child's hospital where they received care for their kidneys
c. Yes, but only temporarily (e.g. friend or relative's home or other temporary housing) because ____________
d. Yes, for other reasons _________________
e. Not applicable to our family

11. What is your family's total estimated income in the last year? (before tax, from all sources including salaries and benefits) Chronic Kidney Disease can have a knock-on effect on a family's circumstances, which is why we would like to understand this better by asking this information.

a. Up to £10,000
b. £10,001 to £20,000
c. £20,001 to £30,000
d. £30,001 to £50,000
e. £50,001 to £70,000
f. £70,001 to £150,000
g. Over £150,000
h. Prefer not to say

12. Next, we would like to ask you questions about your child's medications as this may have an effect on their quality of life.
Please select all of the kidney medicines your child is taking at the moment from this menu

- Aciclovir
- Azathioprine
- Calcium Acetate
- Calcium Carbonate
- Captopril
- Cefaclor
- Cefixim
- Ciclosporin (Sandimunn)
- Co-trimoxazole

For peer review only - http://bmjopen.bmj.com/site/about/guidelines.xhtml

- Darbepoetin
- Enalapril
- Epoetin
- Everolimus (Certican)
- Ganciclovir
- Growth Hormone
- Irbesartan
- Iron (Ferrous fumarate, Ferrous sulphate)
- Lactulose
- Losartan
- Movicol
- Mycophenolate Mofetil (MMF) (Cellcept, Myfenax)
- Nitrofurantoin
- Potassium Chloride
- Potassium Citrate
- Prednisolone
- Senna
- Sevelamer
- Sirolimus (Rapamune)
- Sodium Bicarbonate
- Sodium Chloride
- Tacrolimus (Advagraf, Prograf, Modigraf)
- Trimethoprim
- Valaciclovir
- Valganciclovir
- Vitamin D (Alfacalcidol)
- Vitamin D (Colecalciferol)
- Other(s)________ ________________

13. Please select how often your child takes these medicines (select all that apply):

a. Longer than every month
b. Every month
c. Every other week
d. Every week
e. Three times a week
f. Every other day
g. Once a day
h. Twice a day
i. Three times a day
j. Four times a day
k. More than four times a day
l. Other regime __________

14. Please select how these medicines are given to your child (select all that apply)

a. An injection directly into their vein (via cannula or other access to their veins)
b. Other type of injection (e.g. into skin or muscle)
c. Tablets (by mouth)
d. Tablets (by gastrostomy tube)
e. Liquids (by mouth)
f. Liquids (by gastrostomy tube)
g. Eye drops
h. Creams / Lotions
i. Inhaler
j. Other type of medicine ____________

For peer review only - http://bmjopen.bmj.com/site/about/guidelines.xhtml

15. **Some families may have had their journey towards a kidney transplant affected by the COVID-19 pandemic. Please select which situation applies to your child and family due to the COVID-19 pandemic in 2020-2021:**
    a. Our family was not in the middle of preparing for a kidney transplant during 2020-2021. Therefore COVID-19 did not impact us.
    b. Our family already started or were starting to prepare for a kidney transplant in 2020-2021. The COVID-19 pandemic did not change our preparation plans.
    c. Our family delayed preparations for a kidney transplant from a living donor due to the COVID-19 pandemic.
    d. Our family came off the waiting list for a kidney transplant from a deceased donor due to the COVID-19 pandemic
    e. Our family delayed investigations that were part of preparing for a kidney transplant overall because of the COVID-19 pandemic
    f. Our family's transplant journey was affected by the COVID-19 pandemic is other ways ___________________________

For peer review only

BMJ Open

Protocol for a multi-centre prospective exploratory mixed-methods study investigating the modifiable psychosocial variables influencing access to and outcomes after kidney transplantation in children and young people in the UK

Journal:	*BMJ Open*
Manuscript ID	bmjopen-2023-078150.R2
Article Type:	Protocol
Date Submitted by the Author:	01-May-2024
Complete List of Authors:	Kim, Ji Soo; Great Ormond Street Hospital for Children NHS Foundation Trust, Paediatric Nephrology; NIHR Great Ormond Street Hospital Biomedical Research Centre<br>Wray, Jo; Great Ormond Street Hospital for Children NHS Foundation Trust, Centre for Outcomes and Experience Research in Children's Health, Illness and Disability<br>Ridout, Deborah; UCL, Population, Policy and Practice, UCL Great Ormond Street Institute of Child Health<br>Plumb, Lucy ; UK Renal Registry; Bristol Medical School, Population Health Sciences<br>Nitsch, Dorothea; London School of Hygiene & Tropical Medicine; UK Renal Registry<br>Robb, Matthew; NHS Blood and Transplant, Statistics and Clinical Studies<br>Paediatric Nephrology (BAPN), British Association for; British Association for Paediatric Nephrology<br>Marks, Dr. Stephen; Great Ormond Street Hospital For Children NHS Trust, Paediatric Nephrology; NIHR Great Ormond Street Hospital Biomedical Research Centre
**Primary Subject Heading**:	Paediatrics
Secondary Subject Heading:	Renal medicine, Qualitative research, Mental health
Keywords:	Renal transplantation < NEPHROLOGY, Paediatric transplant surgery < PAEDIATRIC SURGERY, Paediatric nephrology < NEPHROLOGY, Health Services Accessibility, Quality of Life, Social Support

SCHOLARONE™
Manuscripts

Protocol for a multi-centre prospective exploratory mixed-methods study investigating the modifiable psychosocial variables influencing access to and outcomes after kidney transplantation in children and young people in the UK

For peer review only

Ji Soo Kim[1,2], Jo Wray[3], Deborah Ridout[4], Lucy Plumb[5,6], Dorothea Nitsch[6,7], Matthew Robb[8], British Association for Paediatric Nephrology, Stephen D Marks[1,2]

1. Department of Paediatric Nephrology, Great Ormond Street Hospital for Children NHS Foundation Trust, Great Ormond Street London, UK
2. NIHR Great Ormond Street Hospital Biomedical Research Centre, University College London Great Ormond Street Institute of Child Health, London, UK
3. Centre for Outcomes and Experience Research in Children's Health, Illness and Disability, Great Ormond Street Hospital for Children NHS Foundation Trust, London, UK
4. Population, Policy and Practice Department, University College London Great Ormond Street Institute of Child Health, London, UK
5. Population Health Sciences, University of Bristol Medical School, Bristol, UK.
6. UK Renal Registry, Bristol, UK
7. Department of Non-communicable disease epidemiology, London School of Hygiene & Tropical Medicine
8. Statistics and Clinical Studies team at NHS Blood and Transplant, Bristol, UK

Address for Correspondence:
Professor Stephen D Marks, Dr Ji Soo Kim
Department of Paediatric Nephrology
Great Ormond Street Hospital for Children NHS Foundation Trust
London
WC1N 3JH
stephen.marks@gosh.nhs.uk jisoo.kim@nhs.net

Word Count: 3698/4000 words (excluding title page, abstract, tables, author's contribution, acknowledgments & references)
Abstract: 298/300 words

For peer review only - http://bmjopen.bmj.com/site/about/guidelines.xhtml

Abbreviations

CKD-5 – Stage V chronic kidney disease

KRT – kidney replacement therapy

MDT – multi-disciplinary team

QoL – Quality of Life

For peer review only

Keywords

Renal transplantation

Paediatric transplant surgery

Paediatric nephrology

Health Services Accessibility

Quality of Life

Social Support

For peer review only - http://bmjopen.bmj.com/site/about/guidelines.xhtml

Abstract

Introduction

Kidney transplantation is the preferred therapy for children with Stage 5 chronic kidney disease (CKD-5). However, there is wide variation in access to kidney transplantation across the UK for children. This study aims to explore the psychosocial factors that influence access to and outcomes after kidney transplantation in children in the UK using a mixed methods prospective longitudinal design.

Methods

Qualitative data will be collected through semi-structured interviews with children affected by CKD-5, their carers and paediatric renal multi-disciplinary team. Recruitment to interviews will continue till data saturation. These interviews will inform the choice of existing validated questionnaires, which will be distributed to a larger national cohort of children with pre-transplant CKD-5 (n≈180) and their carers. Follow-up questionnaires will be sent at protocolised time points regardless of whether they receive a kidney transplant or not. Co-existing health data from hospital, UK renal registry and NHS Blood and Transplant registry records will be mapped to each questionnaire time point. An integrative analysis of the mixed qualitative and quantitative data will define psychosocial aspects of care for potential intervention to improve transplant access.

Analysis

Qualitative data will be analysed using thematic analysis. Quantitative data will be analysed using appropriate statistical methods to understand how these factors influence access to transplantation, as well as the distribution of psychosocial factors pre- and post-transplantation.

Ethics and Dissemination

This study protocol has been reviewed by the National Institute for Health Research Academy and approved by the Wales Research Ethics Committee 4 (IRAS number 270493/ref: 20/WA/0285) and the Scotland A Research Ethics Committee (ref: 21/SS/0038). Results from this study will be

For peer review only - http://bmjopen.bmj.com/site/about/guidelines.xhtml

disseminated across media platforms accessed by affected families, presented at conferences, and published in peer-reviewed journals.

Registration Details

This work is supported by the National Institute for Health Research Academy, grant number NIHR300727.

For peer review only

For peer review only - http://bmjopen.bmj.com/site/about/guidelines.xhtml

Article Summary:

Strengths and limitations of this study

1. Prospective, longitudinal study design allows for a more detailed understanding of delays in transplantation associated with different psychosocial factors.
2. Combination of qualitative and quantitative data enable in-depth exploration of how psychosocial factors influence access to transplantation and outcomes thereafter.
3. Involvement of the majority of UK paediatric nephrology units ensuring representation of paediatric CKD-5 population
4. Limited follow-up timeline will capture only short to medium-term rather than long-term outcomes.
5. Utilisation of interpreters in qualitative interviews increases involvement of non-English speaking families, however the lack of validated translations of questionnaires may limit involvement of non-English speaking families in capturing quantitative data.

For peer review only

For peer review only - http://bmjopen.bmj.com/site/about/guidelines.xhtml

Introduction

Around 1,000 children (age 0-17 years) with Stage 5 chronic kidney disease (CKD-5) in the United Kingdom receive Kidney Replacement Therapy (KRT) in the form of either peritoneal dialysis, haemodialysis or kidney transplantation[1, 2]. Kidney transplantation is the gold standard therapy for reducing mortality and improving outcomes for children[3]. Minimising time on dialysis in favour of transplantation has been shown to reduce CKD-5 related complications and morbidity [4]. Compared with dialysis, transplantation is also presumed to improve patients' health-related quality of life (HR-QoL)[5]. Furthermore in the UK, for every year that the patient's kidney transplant functions, transplantation is three times more cost-effective than dialysis for the NHS[6].

However, not every child with CKD-5 can access kidney transplantation. There are approximately 193 to 217 prevalent children on dialysis each year[7, 8]. Annually only 130-160 paediatric kidney transplants are performed in the UK[9]. There appears to be variation in transplantation access, practice, and outcomes between UK paediatric nephrology units[1, 2, 9]. A cross-sectional survey conducted by the British Association for Paediatric Nephrology examined reasons for paediatric kidney transplantation delay in the UK. The survey showed that psychosocial factors make up 19% of the barriers, although specific factors were not identified in this study[10]. Compared with paediatrics, considerable research has been done in adult patients, regarding psychosocial factors implicated in transplant access. Formalised pre- and post-transplant psychosocial assessments have been widely researched for adult solid organ transplant recipients[11-14]. The UK-wide study 'Access to Transplantation and Transplant Outcome Measures' specifically investigated psychosocial barriers in adult kidney transplant recipients[15]. Researchers found inequities in transplant access, in spite of a universal healthcare system, based on socioeconomic status, education level, health literacy and racial background[16, 17]. In terms of outcomes, they found no difference in post-transplant HR-QoL between living and deceased donor recipients and that recipient expectations influenced post-transplant recovery[15-19]. However, for UK children, it is less clear what these 'psychosocial factors' are and how they influence unit-specific decisions, access to kidney transplantation and outcomes [10, 20, 21]. We acknowledge the recent progress

For peer review only - http://bmjopen.bmj.com/site/about/guidelines.xhtml

being made in exploring these psychosocial factors in some countries[22-24]. However, psychosocial studies for children with CKD-5 are still limited by retrospective or cross-sectional design or by single-centre or small study cohorts. There are no UK studies that prospectively explore how these factors impact paediatric kidney transplantation access over time.

Aims and Objectives:

This study aims to prospectively evaluate the psychosocial factors that are actual or perceived barriers to paediatric kidney transplantation which may be associated with poor transplant outcome. We anticipate these psychosocial factors to be broad at an individual, family and societal level and that they will encompass mental health and social determinants of health as defined by Marmot: 'the conditions in which people are born, grow, live, work and age'[25]– we hypothesise that psychosocial factors implicated in transplant access can be quantified using formal, validated measures, and that these factors may influence outcomes in the short-term period following transplantation.

This will be achieved through the following research objectives:

1. Describe the psychosocial factors perceived by clinicians as barriers to kidney transplantation.
2. Describe current interventions (if any) implemented to address these psychosocial factors.
3. Explore the experiences and beliefs of children and their families of any psychosocial challenges or facilitators in accessing a kidney transplant
4. Quantify the identified psychosocial factors implicated in CKD management
5. Measure the prevalence of identified psychosocial factors (positive and negative) in the national cohort of patients being pre-emptively worked up for transplant, on dialysis or listed for transplant.
6. Examine these psychosocial factors regarding their association with time to transplant and their changes following transplantation

For peer review only - http://bmjopen.bmj.com/site/about/guidelines.xhtml

7. Synthesise findings from each phase to inform recommendations about which psychosocial factors are potential barriers or facilitators to accessing kidney transplantation.

Findings synthesised from this study will inform development of a complex intervention to improve uptake of kidney transplantation in children with CKD-5 who would benefit most from one.

Methods and Analysis

Study Design Overview:

This prospective study has three phases, commencing with a sequential exploratory mixed-methods design, followed by a sequential explanatory mixed-methods design (Figure 1)[26]. This approach was chosen to first gain new insights by becoming familiar with the range of potential psychosocial factors and then longitudinally observing the influence of these factors on kidney transplantation access and outcomes for children[27]. Phase one will consist of exploratory interviews with purposively selected participants. These interviews will inform which questionnaires will be distributed at baseline and follow-up to the wider cohort of children with CKD-5 and their carer(s) in phase two stage A. Using the interviews to inform questionnaire selection will ensure questionnaires are relevant to, and resonate with, families. Participant families with outlier findings from phase two stage A will then be invited for explanatory interviews in phase two stage B. Finally, in phase three, the qualitative and quantitative data from the previous phases will be analysed together to develop an integrated understanding of psychosocial factors that influence access to kidney transplantation.

Patient and Public Involvement:

For peer review only - http://bmjopen.bmj.com/site/about/guidelines.xhtml

All elements of the study design were co-developed and approved by our Research Partner Family and study steering group. Our Research Partner Family (a young person and parent dyad) and steering group members have lived experience of CKD, dialysis and kidney transplantation in childhood either as a patient or carer. The Great Ormond Street Hospital Young Persons Advisory Group have also approved the research question and study design. The Young Persons Advisory Group consists of children affiliated with Great Ormond Street Hospital for Children NHS Foundation Trust as a patient or family member of a patient and therefore who have the lived experience needed to advise researchers designing studies involving children.

Population:

This study aims to include children, carer and staff participants from all 13 UK paediatric nephrology units, of which 10 units offer kidney transplantation surgery on site and 3 units which do not offer transplantation surgery at their own site but offer shared transplant care with nearby units.

In phase one and phase two stage A, children (aged 0 – 17 years inclusive) with CKD-5 on chronic dialysis, being worked up for pre-emptive kidney transplantation or on the waiting list for deceased donation (active or suspended) or awaiting living donation, will be invited to participate with their carer(s). Where appropriate, children aged 5 years and above will be invited to directly participate. For children aged under 5 years or those who are unable to assent or consent, their carers will be consulted and offered an opportunity to participate through proxy measures. Members of the multi-disciplinary team (MDT) involved in pre-transplantation work-up at their local unit (e.g., paediatric nephrologists, transplant surgeons, nurse specialists, social workers, family therapists, play therapists and members of the psychosocial team etc.) will be invited for interviews in Phase One only. Patients or carer(s) who are unable to give informed consent or patients who have been deemed too unwell to participate or had a recent acute hospital admission in the last 14 days will not be approached to participate in the study.

For peer review only - http://bmjopen.bmj.com/site/about/guidelines.xhtml

Phase One Interviews:

A purposive sampling matrix will be used to maximise the diversity of views. Criteria for sampling will include age, sex, and ethnicity of children, modality of KRT, whether the kidney unit offers transplantation surgery or not and role in the MDT. Completed interviews will be analysed in parallel with ongoing recruitment. Participant recruitment will stop when no new themes are generated from the data.

Analysis of these interviews will contribute towards selecting questionnaires for use in phase two stage A.

Phase Two Stage A Questionnaires:

In this phase, we will aim to recruit every child with CKD-5 who meets the inclusion criteria at pre-transplant baseline with an anticipated recruitment rate of 60-70%.

At the time of study design, the predicted annual numbers across all 13 UK paediatric nephrology units as per UK Renal Registry (UKRR) reports were as follows[1, 2]:

- Number of dialysis patients on the transplant waiting-list in a year; n=70
- Number of patients starting dialysis in a year; n=125
- Number of patients not on dialysis but pre-emptively on the transplant waiting list; n=50
- Transplant rate; 130 to 160 patients per year

Based on these numbers, the predicted maximum number of eligible participants in a 1-year-period would be 245. We chose a 2-year-period for our sample size calculation, which would be a population size of 490, to include children who experience delays and receive their transplant after 1 year (See Figure 2). We plan to recruit 180 patients and after allowing for a 20% drop out, we expect to include 141 families. Assuming a prevalence of pertinent psychosocial factors in the national cohort of patients being pre-emptively worked up for transplant, on dialysis or listed for

For peer review only - http://bmjopen.bmj.com/site/about/guidelines.xhtml
For peer review only

transplant of 15%, then with 141 families we will be able to estimate this with 5% precision. At the time of sample size calculation, there were no paediatric studies specifically measuring this nor studies encompassing all psychosocial factors. Therefore, we used the assumed prevalence of 15% based on a study measuring psychological distress in potential adult transplant candidates[28].

We expect some overlap in the cohort between Phase One and Phase Two Stage A and appreciate some families may develop research fatigue. Families who already took part in Phase One will be asked whether they would like to also take part in Phase Two Stage A or prefer to opt out.

Phase Two Stage B:

Results from phase two stage A will be reviewed for negative and positive cases. Families who have outlier findings, in terms of their answers to the validated questionnaires, will be invited to interview in phase two stage B, with the aim of reviewing and refining themes to develop additional theoretical explanation to the influence of psychosocial factors on transplant access and outcomes. Exact parameters to define outliers and sample size will be dependent on findings from phase two stage A.

Outcomes measured:

Phase One:

Using a Topic Guide (Supplementary Material 1), qualitative data will be collected in phase one to address research objectives 1-4 through semi-structured, in-depth interviews undertaken by the investigator (JSK). This format was chosen over focus groups to enable participation of younger or less verbal children and avoid further inconveniencing families through multiple research appointments from having separate interviews for young children and focus groups for carers. The family interview format will be based on the childs' decision to either interview with or separately from their carers, depending on which setting they find more comfortable. To ensure no participant is unfairly excluded from interviews due to English not being their first language, interpreters will

For peer review only - http://bmjopen.bmj.com/site/about/guidelines.xhtml

be present to support their participation. The same principle of minimising communication barriers will be applied for younger children or young people who may prefer communicating through other creative outputs such as drawing or Talking Mats. The research goals for family interviews will include exploring what families feel their life is like now living with CKD-5, what good HR-QoL looks like and what they believe delays or enables how soon they receive a kidney transplant. Similarly, research goals for MDT member interviews will include exploring what the professionals think matters most to families whose child has CKD-5 when it comes to a good HR-QoL and what they, as professionals, believe impacts how soon children access a kidney transplant in terms of psychosocial factors.

The Topic Guide has been developed from the investigator's systematic literature review on the subject matter and in consultation with the Young Persons Advisory Group and the Research Partner Family[29]. To minimise any inconvenience for joining the study, all participants will be interviewed using their preferred modality (telephone, video-call or face-to-face consultations) in keeping with COVID-19 safety recommendations[30]. Interview time with young children will be kept to less than half an hour to minimise interview fatigue. All interviews will be audio or video recorded depending on participant preference.

Participants may become distressed as they reflect on their experiences due to the sensitive nature of some of the interview topics around their mental health or transplant delays. Therefore, the participant and JSK will agree on a 'stop signal' before commencing the interview for use should they feel uncomfortable. If the participant uses the 'stop signal', they will be offered a break. The participant and JSK will then discuss whether they would like to continue, reschedule or withdraw from the interview altogether. Once the interview recording stops, there will be an opportunity to discuss the participant's feelings and if appropriate, they will be signposted to their local support services.

The acceptability of existing validated age-appropriate questionnaires that measure outcomes relevant to the preliminary themes, will be discussed with the steering group. Potential validated questionnaires that capture the preliminary themes will be identified from the systematic literature review and a wider search of the literature. These questionnaires will be checked in terms of their

For peer review only - http://bmjopen.bmj.com/site/about/guidelines.xhtml

psychometric properties such as internal consistency (Cronbach alpha of at least 0.7) and test re-test reliability and aspects such as availability of the measures, respondent type (parent, child, or other respondent) and age range for which the questionnaire has been validated, to enable the most appropriate questionnaire to be chosen to measure each theme. The selected questionnaires will then be discussed with the steering group, considering the language of the questionnaire, acceptability, and level of burden for the participant. If the list of questionnaires is too onerous for the participating family, a consensus will be reached with the steering group on which preliminary themes and therefore which questionnaires should be prioritised. Once the final list of questionnaires is agreed on, these will be submitted to the Health Research Authority for final approval.

Phase Two Stage A:

In phase two stage A, research objectives 5-6 will be addressed by measuring the following primary outcome variables: HR-QoL, psychosocial functioning and time taken to receive a kidney transplant since date confirming CKD-5. To understand changes in psychosocial factors over time, their associations with health burden or short-term allograft deterioration must be accounted for, Therefore, we will measure the following secondary outcome: the child's estimated glomerular filtration rate (eGFR) over time. The eGFR will be calculated with their height and serum creatinine using the CkiD U25 formula [31, 32].

Questionnaires measuring HR-QoL and psychosocial functioning, selected from phase one, will be distributed to a larger, national cohort of children with CKD-5 and their carers at their pre-transplant baseline. Follow-up questionnaires will be sent post-transplant at 3-, 6- and 12-months later or 12-months after their first questionnaire if they still have not received a kidney transplant in that time frame. These follow-up time points were chosen to reflect the initial period of post-transplant adaptation, which are comparable with similar studies in adults and children at the time of protocol-writing[33-35]. For children who have not received a transplant, a 12-month interval was advised by our Steering Group to avoid distress triggered by frequent questionnaires

For peer review only - http://bmjopen.bmj.com/site/about/guidelines.xhtml

reminding them of their non-transplanted state. As families are more likely to participate if they can choose which questionnaire modality is most suited to their lifestyle, participant families will be offered either paper or online questionnaires[36]. Health morbidity and co-existing disease data, including their underlying primary kidney diagnosis, as described in Table 1, will be mapped to each questionnaire time point.

Baseline of all participating CYP	CYP not transplanted yet at 12 months follow-up	CYP who are Post-transplant
• Ethnicity<br>• Primary renal diagnosis<br>• Other co-morbidity diagnoses<br>• Modality of KRT if applicable<br>• Date confirming CKD-5<br>• Date of starting RRT if applicable<br>• Date of previous kidney transplant reaching CKD-5 if applicable<br>• Height (cm)<br>• Weight (kg)<br>• Serum Creatinine (ummol/L)<br>• Number and type of medications<br>• Post code*	• Modality of KRT if changed (if applicable)<br>• Date of starting new modality of RRT (if applicable)<br>• Height (cm)<br>• Weight (kg)<br>• Serum Creatinine (ummol/L)<br>• Number and type of medications*	Only at 3 months post-transplant<br>• Type of transplant kidney (Living related / Living Unrelated / Deceased Donor (after cardiac or brain death)<br>• Donor & recipient mismatch<br>• ABO compatibility<br>• Cold ischaemic Time<br>• Need for plasma exchange / immunoadsorption / Rituximab / iv immunoglobulin<br>At all post-transplant intervals (3-, 6-, 12- and 24-months)<br>• Changes to co-morbidities if applicable<br>• Change of KRT modality if applicable<br>• Date of RRT modality change if applicable<br>• Height (cm)<br>• Weight (kg)<br>• Serum Creatinine (ummol/L)<br>• Number and type of medications

Table 1: Co-existing Health Data to be collected and mapped against questionnaire time points

** Post code data will be converted to the relevant national multiple indices of deprivation (e.g. English Indices of Deprivation, Scottish Index of Multiple Deprivation)*

An additional questionnaire (see Supplementary Material 2) has been co-designed with the steering group to retrieve information on the participating family's demographic background. Data on medication burden will also be collected due to its' possible confounding effect HR-QoL as described in current literature[37].

For peer review only - http://bmjopen.bmj.com/site/about/guidelines.xhtml

Phase Two Stage B:

The Interview Topic Guide for Phase Two Stage B has been designed with flexibility, since the interview will depend on the nature of the outlier findings from Phase Two Stage A.

Data Analysis Plan:

Phase One:

To address research objectives 1-4, JSK will thematically analyse interview transcripts, following the approach of Braun and Clarke [38]. De-identified transcripts will be managed using Nvivo software[39]. For the purposes of qualitative rigour, JSK will maintain a reflexivity journal after interviews and throughout data analysis, to ensure potential researcher biases and insights are noted. Maintaining a reflexivity journal is a gold standard practice in qualitative research, which increases the credibility and deepens the understanding of the findings by describing the context in which data were collected and analysed[40, 41]. A sample of transcripts will be examined by JW and discussed with JSK. Throughout all stages of data collection and analysis, findings will be shared and discussed by JSK, JW and SDM.

Phase Two Stage A:

Participant demographics will be described using descriptive statistics. It will also be used to address research objective 4 by describing the prevalence of psychosocial factors.

Research objective 6 will be addressed through the following:

For peer review only - http://bmjopen.bmj.com/site/about/guidelines.xhtml

First, descriptive statistics will again be utilised to describe how participants' psychosocial functioning and HR-QoL variables change before and after transplantation at each data collection time point.

Second, to describe the impact waiting to access a kidney transplant and receiving one has on psychosocial functioning and HR-QoL over time, repeated measure ANOVA will be undertaken.

Third, the association between clinical, demographic, and psychosocial factors with accessing a kidney transplant will be measured using logistic regression modelling and Kaplan-Meier statistics.

Fourth, the association of clinical, demographic, and psychosocial factors with failing transplant allograft in short-term follow-up between one- and two-years post-transplantation will be assessed. Where the outcome variable is the child's eGFR, linear regression modelling will be used. Where the outcome variable defines a failing post-transplant kidney as reaching an eGFR equivalent to stage IV CKD (eGFR 15-29 ml/min/1.73m^2), logistic regression modelling and Kaplan-Meier statistics will be utilised.

Separately, the level of agreement between child and carer responses will be assessed. Choice of statistical tests will depend on the type of data: for example, Cohen's Kappa for binary data, weighted Kappa for ordinal data or interclass correlation coefficients for continuous data.

Where relevant, all statistical analyses will be adjusted for (but not limited to) child's age, gender, ethnicity, socioeconomic status, and KRT status at baseline.

For peer review only

Phase Two Stage B:

Interview data will be analysed using Thematic Analysis underpinned by the same principles as in phase one.

Phase Three:

For peer review only - http://bmjopen.bmj.com/site/about/guidelines.xhtml

An integrative analysis of data from phases one and two will be undertaken to address research objective 7 and create a conceptual model of how psychosocial factors influence transplant access and outcomes. The quantitative and qualitative data will be reviewed together to understand where they converge, complement, or diverge from the other dataset or have findings that are novel. A final lay report will be co-written with the steering group to ensure it is meaningful, relevant, and accessible to families whose child have CKD.

Ethics

This study protocol has been peer reviewed by the National Institute for Health Research Academy and has been approved under IRAS number 270493 by the Wales Research Ethics Committee 4 (ref: 20/WA/0285) and the Scotland A Research Ethics Committee (ref: 21/SS/0038).

Coercive pressure of joining the study will be minimised. Research participants will not be paid for participation. Participant information sheets will indicate that there will be no added benefit or disruption to their medical care.

Informed written consent will be obtained from all participants aged ≥16 years old and written assent from participants aged 5 – 15 years old. Consent will be obtained for all research activities including interviews, questionnaires and retrieving health information from national databases and hospital records.

Participant confidentiality will be upheld by fully adhering to the Data Protection Act. Participant identifiers will be handled with appropriate pseudonymisation and all data will be kept on GDPR-compliant encrypted devices or stored on hard drives with restricted access with the relevant encryption and password protection. The only instance where confidentiality will be breached is if a participant discloses information that has direct implications for child or adult safeguarding. Potential participants will be made aware of this as part of informed consent. All interviews will be

For peer review only - http://bmjopen.bmj.com/site/about/guidelines.xhtml

digitally recorded, transcribed verbatim and have identifiable data redacted. Audio files will be transcribed either by JSK or by a third-party interview transcription company (Take Note®). To minimise data handling breaches, all engagement with Take Note® will only be through their secure web platform. All video files will be transcribed only by JSK to remove third party involvement in the de-identification process. Once de-identified, only quotes that cannot lead to participant identification will be used in reports. Care will be taken in reporting findings to ensure individuals cannot be identified by their role, diagnosis, gender, age or geographic locality.

Dissemination

Research participants will be updated about the findings through newsletters. Lay summaries approved by the steering group will be disseminated across charity websites accessed by children and families affected by CKD. To ensure that professionals who work with families affected by CKD are being reached, findings will be disseminated widely in relevant peer-reviewed journals and at national and international conferences. Finally, if appropriate, a dissemination strategy will be co-created between JSK and the steering group for other professionals (e.g., teachers) who may encounter vulnerable families with CKD and need early referral for psychosocial intervention.

For peer review only

For peer review only - http://bmjopen.bmj.com/site/about/guidelines.xhtml

Author's contribution

- JSK wrote the study protocol and coordinated the entire manuscript.
- Deborah Ridout contributed towards sample size calculation and statistical methods of study protocol.
- LP contributed towards statistical analysis of protocol and advised regarding UK Renal Registry involvement.
- DN contributed towards statistical analysis of protocol and advised regarding UK Renal Registry involvement.
- MR contributed towards statistical analysis of protocol and advised regarding NHS Blood & Transplant involvement.
- BAPN contributed towards study protocol development in terms of multi-centre data collection feasibility.
- JW supervised JSK, study protocol development with regular input to initial drafts and final manuscript approval.
- SDM supervised JSK, study protocol development with regular input to initial drafts and final manuscript approval.

For peer review only

Acknowledgements

We would like to thank the Great Ormond Street Hospital Young Person's Advisory Group and members of our Research Partner Family Steering Group –Emma Beeden, Katy Beeden, Angela Watt and Heather Davis from KDARS (Kidney Disease and Renal Support) for kids, for their expertise, support and advice. Finally, we would also like to thank members of the British Association for Paediatric Nephrology, the National Kidney Federation and Kidney Care UK for their continued support of this study.

Funding Statement:

This work is supported by the National Institute for Health Research Academy, as a Doctoral Fellowship, grant number NIHR300727.

Competing Interests Statement:

Dr Ji Soo Kim is the National Institute for Health Research Fellowship grant recipient, which funds this study.

Dr Lucy Plumb reports grants from the National Institute for Health Research and Kidney Research UK. She is also the paediatric research lead for the UK Renal Registry.

Figure Legends

Figure 1: Flow diagram of sequential mixed method study design

Figure 2: Flow diagram illustrating predicted number of CYP who are eligible for transplantation and receive one across two years

For peer review only

References

1. Plumb L, Casula A, Pyart R, Evans KM, Inward C, Medcalf J, et al. The 21st UK Renal Registry Annual Report: A Summary of Analyses of Paediatric Data in 2017. Nephron. 2019:1-5.
2. Plumb L, Wong E, Casula A, Braddon F, Lewis M, Marks SD, et al. Chapter 4 Demography of the UK Paediatric Renal Replacement Therapy Population in 2016. Nephron. 2018;139 Suppl 1:105-16.
3. Wolfe RA, Ashby VB, Milford EL, Ojo AO, Ettenger RE, Agodoa LYC, et al. Comparison of Mortality in All Patients on Dialysis, Patients on Dialysis Awaiting Transplantation, and Recipients of a First Cadaveric Transplant. New England Journal of Medicine. 1999;341(23):1725-30.
4. Pruthi R, Casula A, Inward C, Roderick P, Sinha MD. Early Requirement for RRT in Children at Presentation in the United Kingdom: Association with Transplantation and Survival. Clin J Am Soc Nephrol. 2016;11(5):795-802.
5. Park KS, Hwang Yj Fau - Cho MH, Cho Mh Fau - Ko CW, Ko Cw Fau - Ha IS, Ha Is Fau - Kang HG, Kang Hg Fau - Cheong HI, et al. Quality of life in children with end-stage renal disease based on a PedsQL ESRD module. 2012(1432-198X (Electronic)).
6. Kerr M, Bray B, Medcalf J, O'Donoghue DJ, Matthews B. Estimating the financial cost of chronic kidney disease to the NHS in England. Nephrol Dial Transplant. 2012;27 Suppl 3:iii73-80.
7. UKRR. UK Renal Registry 21st Annual Report - data to 31/12/2017 Bristol, UK2019 [cited 2023. Available from: https://www.renalreg.org/publications-reports/.
8. UKRR. UK Renal Registry 24th Annual Report - data to 31/12/2020 Bristol, UK2022 [Available from: https://ukkidney.org/audit-research/annual-report.
9. Transplant OD. Annual Report on Kidney Transplantation Report for 2016/2017 NHS Blood and Transplant2017 [Available from: https://nhsbtdbe.blob.core.windows.net/umbraco-assets-corp/4607/kidney-annual-report-2016-17.pdf.
10. Kim JS, Marlais M, Balasubramanian R, Muorah M, Inward C, Smith GC, et al. UK national study of barriers to renal transplantation in children. Archives of disease in childhood. 2020.
11. Feurer ID, Moore DE, Speroff T, Liu H, Payne J, Harrison C, et al. Refining a health-related quality of life assessment strategy for solid organ transplant patients. Clin Transplant. 2004;18 Suppl 12:39-45.
12. Goetzmann L, Ruegg L, Stamm M, Ambuhl P, Boehler A, Halter J, et al. Psychosocial profiles after transplantation: a 24-month follow-up of heart, lung, liver, kidney and allogeneic bone-marrow patients. Transplantation. 2008;86(5):662-8.
13. Maldonado JR, Sher Y, Lolak S, Swendsen H, Skibola D, Neri E, et al. The Stanford Integrated Psychosocial Assessment for Transplantation: A Prospective Study of Medical and Psychosocial Outcomes. Psychosom Med. 2015;77(9):1018-30.
14. Shah VS, Ananth A, Sohal GK, Bertges-Yost W, Eshelman A, Parasuraman RK, et al. Quality of life and psychosocial factors in renal transplant recipients. Transplant Proc. 2006;38(5):1283-5.
15. Wu DA, Robb ML, Watson CJE, Forsythe JLR, Tomson CRV, Cairns J, et al. Barriers to living donor kidney transplantation in the United Kingdom: a national observational study. Nephrol Dial Transplant. 2017;32(5):890-900.
16. Pruthi R, Robb ML, Oniscu GC, Tomson C, Bradley A, Forsythe JL, et al. Inequity in Access to Transplantation in the United Kingdom. Clin J Am Soc Nephrol. 2020;15(6):830-42.
17. Taylor DM, Bradley JA, Bradley C, Draper H, Dudley C, Fogarty D, et al. Limited health literacy is associated with reduced access to kidney transplantation. Kidney Int. 2019;95(5):1244-52.

For peer review only - http://bmjopen.bmj.com/site/about/guidelines.xhtml

18. Wu DA, Robb ML, Forsythe JLR, Bradley C, Cairns J, Draper H, et al. Recipient Comorbidity and Survival Outcomes after Kidney Transplantation: a UK-wide Prospective Cohort Study. Transplantation. 2019.
19. Gibbons A, Bayfield J, Cinnirella M, Draper H, Johnson RJ, Oniscu GC, et al. Changes in quality of life (QoL) and other patient-reported outcome measures (PROMs) in living-donor and deceased-donor kidney transplant recipients and those awaiting transplantation in the UK ATTOM programme: a longitudinal cohort questionnaire survey with additional qualitative interviews. BMJ Open. 2021;11(4):e047263.
20. Fung E, Shaw RJ. Pediatric Transplant Rating Instrument - a scale for the pretransplant psychiatric evaluation of pediatric organ transplant recipients. Pediatr Transplant. 2008;12(1):57-66.
21. Killian MO, Schuman DL, Mayersohn GS, Triplett KN. Psychosocial predictors of medication non-adherence in pediatric organ transplantation: A systematic review. Pediatr Transplant. 2018;22(4):e13188.
22. Anand A, Malik TH, Dunson J, McDonald MF, Christmann CR, Galvan NTN, et al. Factors associated with long-term graft survival in pediatric kidney transplant recipients. Pediatr Transplant. 2021;25(4):e13999.
23. Freiberger D, Kimball B, Traum AZ, Berbert L, O'Melia L, Daly KP, et al. Equity factors in pediatric transplant listing: Initial findings from a single center review. Pediatr Transplant. 2023;27(2):e14467.
24. Rea KE, West KB, Dorste A, Christofferson ES, Lefkowitz D, Mudd E, et al. A systematic review of social determinants of health in pediatric organ transplant outcomes. Pediatr Transplant. 2023;27(1):e14418.
25. Marmot MA, T; Bell, J; Black, C; Broadfoot, P; Cumberlege, J; Diamond, I; Gilmore, I; Ham, C; Meacher, M; Mulgan, G. Fair Society, Healthy Lives, The Marmot Review: Institute of Health Equity; 2010 [Available from: https://www.instituteofhealthequity.org/resources-reports/fair-society-healthy-lives-the-marmot-review.
26. Creswell JW. A Concise Introduction to Mixed Methods Research. 1 ed. Thousand Oaks, United States: SAGE Publications Inc; 2014.
27. Reiter B. Theory and Methodology of Exploratory Social Science Research. International Journal of Science and Research Methodology 2017;5(4):129-50.
28. Kuntz KK, Bonfiglio DB. Psychological distress in patients presenting for initial renal transplant evaluation. J Clin Psychol Med Settings. 2011;18(3):307-11.
29. Kim J, Chesters H, Wray J, Marks SD. What are the psychosocial factors that influence access to kidney transplantation (and their outcomes) for children and young people? A Systematic Literature Review Protocol University of York: PROSPERO; 2020 [cited 2021. Available from: https://www.crd.york.ac.uk/PROSPEROFILES/210506_PROTOCOL_20210413.pdf.
30. BAPN. COVID-19 Updated Shielding Guidance for Children and Young People with Kidney Disease UK: Renal Association; 2020 [cited 2020. Available from: https://renal.org/wp-content/uploads/2020/06/Shielding-guidance-25.06.2020.pdf.
31. Ng DK, Pierce CB. Kidney Disease Progression in Children and Young Adults With Pediatric CKD: Epidemiologic Perspectives and Clinical Applications. Semin Nephrol. 2021;41(5):405-15.
32. Pierce CB, Muñoz A, Ng DK, Warady BA, Furth SL, Schwartz GJ. Age- and sex-dependent clinical equations to estimate glomerular filtration rates in children and young adults with chronic kidney disease. Kidney Int. 2021;99(4):948-56.
33. Kugler C, Gottlieb J, Warnecke G, Schwarz A, Weissenborn K, Barg-Hock H, et al. Health-related quality of life after solid organ transplantation: a prospective, multiorgan cohort study. Transplantation. 2013;96(3):316-23.
34. Wightman A, Bradford MC, Smith J. Health-related quality of life changes following renal transplantation in children. Pediatr Transplant. 2019;23(2):e13333.
35. Wyld M, Morton RL, Hayen A, Howard K, Webster AC. A systematic review and meta-analysis of utility-based quality of life in chronic kidney disease treatments. PLoS Med. 2012;9(9):e1001307.

For peer review only - http://bmjopen.bmj.com/site/about/guidelines.xhtml

36. Pulham RA, Wray J, Feinstein Y, Brown K, Pierce C, Nadel S, et al. Feasibility and Acceptability of Methods to Collect Follow-Up Information From Parents 12 Months After Their Child's Emergency Admission to Pediatric Intensive Care. Pediatr Crit Care Med. 2019;20(4):e199-e207.
37. Díaz-González de Ferris ME, Pierce CB, Gipson DS, Furth SL, Warady BA, Hooper SR. Health-related quality of life in children with chronic kidney disease is affected by the number of medications. Pediatr Nephrol. 2021;36(5):1307-10.
38. Braun V, Clarke V. Using thematic analysis in psychology. Qualitative Research in Psychology. 2006;3(2):77-101.
39. Lumivero. NVivo Denver2020 [cited 2023 2023]. 2020:[Available from: https://lumivero.com/products/nvivo/.
40. Dodgson JE. Reflexivity in Qualitative Research. J Hum Lact. 2019;35(2):220-2.
41. Russell GM, Kelly NH. Research as Interacting Dialogic Processes: Implications for Reflexivity. Forum Qualitative Sozialforschung / Forum: Qualitative Social Research. 2002;3(3).

For peer review only

For peer review only - http://bmjopen.bmj.com/site/about/guidelines.xhtml

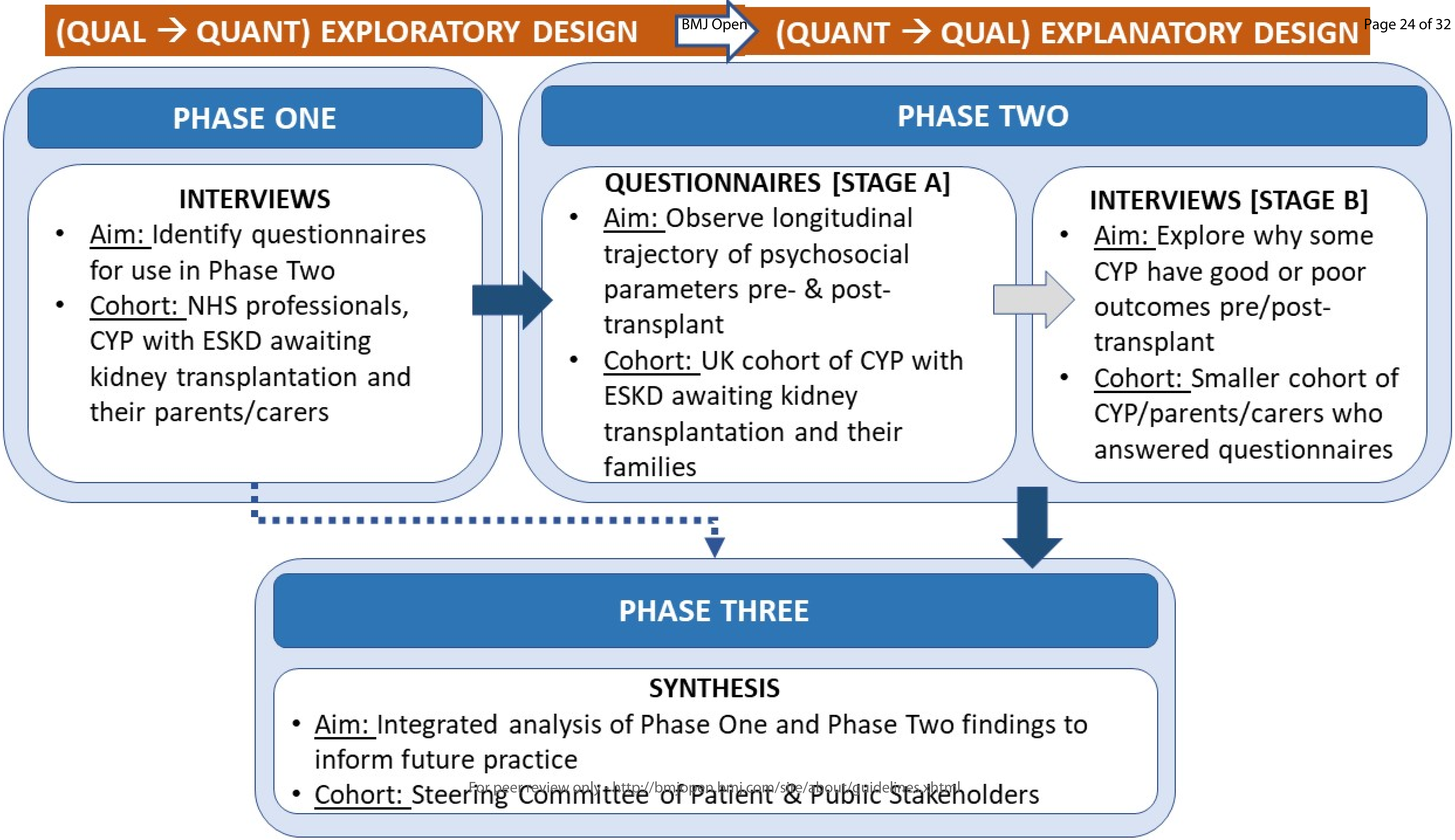
(QUAL → QUANT) EXPLORATORY DESIGN
(QUANT → QUAL) EXPLANATORY DESIGN
PHASE ONE
INTERVIEWS
• Aim: Identify questionnaires for use in Phase Two
• Cohort: NHS professionals, CYP with ESKD awaiting kidney transplantation and their parents/carers
PHASE TWO
QUESTIONNAIRES [STAGE A]
• Aim: Observe longitudinal trajectory of psychosocial parameters pre- & post-transplant
• Cohort: UK cohort of CYP with ESKD awaiting kidney transplantation and their families
INTERVIEWS [STAGE B]
• Aim: Explore why some CYP have good or poor outcomes pre/post-transplant
• Cohort: Smaller cohort of CYP/parents/carers who answered questionnaires
PHASE THREE
SYNTHESIS
• Aim: Integrated analysis of Phase One and Phase Two findings to inform future practice
• Cohort: Steering Committee of Patient & Public Stakeholders

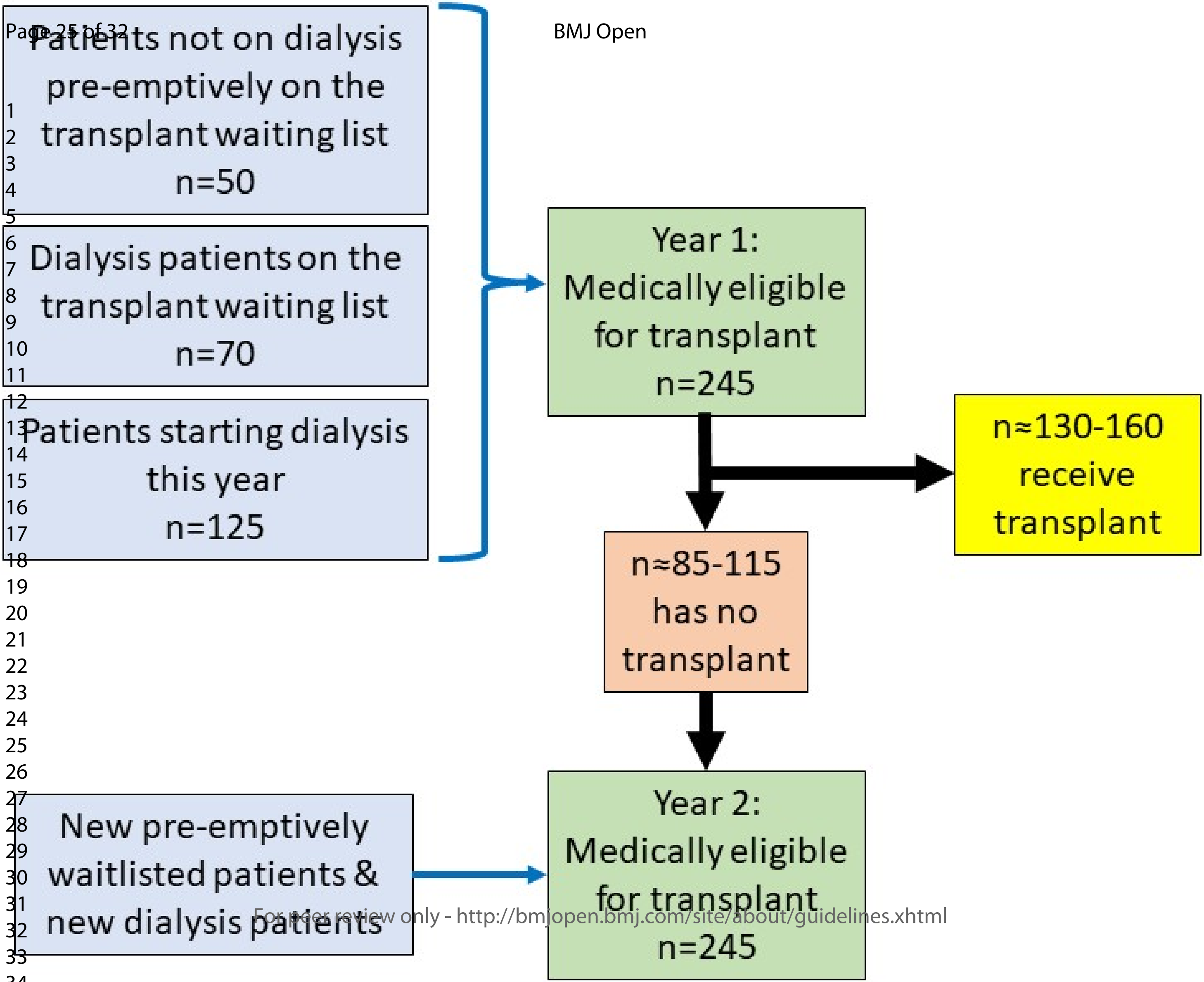
Patients not on dialysis pre-emptively on the transplant waiting list n=50
Dialysis patients on the transplant waiting list n=70
Patients starting dialysis this year n=125
Year 1: Medically eligible for transplant n=245
n≈130-160 receive transplant
n≈85-115 has no transplant
New pre-emptively waitlisted patients & new dialysis patients
Year 2: Medically eligible for transplant n=245

Supplementary Material 1

An investigation of the modifiable psychosocial variables influencing access to and outcomes after kidney transplantation in children

Interview Topic Guide for Researcher – with parent / guardian

Research Goals of the Interview:

1. Explore how the participant feels their life is now (with End Stage Kidney Disease [ESKD])
2. Explore what good quality life looks like to the participant – what matters most?
3. Explore what the participant believes is delaying or enabling their child to receive a kidney transplant

[1] Introduction, Overview:

- *Welcome, introduction of the research project and the researcher*
- *Establish a 'stop sign' for research participant to use if needing to pause the interview*

[2] Discussion of…:

Note for Interviewer: Subjects covered in this topic guide are to be explored openly – not leading. Appropriate prompts will be used – e.g. can you tell me more about that? How did that make you feel?

1. What is day-to-day living with ESKD like? (exploring different aspects of daily life – family life, school/college, employment, any treatment, limitations, participation in activities etc)
2. Which aspects of daily-living really matter to a family living with ESKD?
3. What are their experiences and beliefs around preparing for/receiving a kidney transplant?

[3] Conclusion:

1. *Summarise what has been discussed, mention the positive details, thank the young person / parent / carer*
2. "How did you find talking about living with chronic kidney disease?"
3. "Is there anything important to YOU that we have not talked about?"
4. Handle any concerns they have, signpost to any local services that could support their concerns.

For peer review only - http://bmjopen.bmj.com/site/about/guidelines.xhtml

An investigation of the modifiable psychosocial variables influencing access to and outcomes after kidney transplantation in children

Interview Topic Guide for Researcher – with the Young Person

Research Goals of the Interview:

4. Explore how the participant feels their life is now (with End Stage Kidney Disease [ESKD])
5. Explore what good quality life looks like to the participant – what matters most?
6. Explore what the participant believes is delaying or enabling the young person to receive a kidney transplant

[1] Introduction, Overview:

- *Welcome, introduction of the research project and the researcher*
- *Establish a 'stop sign' for research participant to use if needing to pause the interview*

[2] Discussion of…:

Note for Interviewer: Subjects covered in this topic guide are to be explored openly – not leading. Appropriate prompts will be used – e.g. can you tell me more about that? How did that make you feel?

4. What is day-to-day living with ESKD like? (exploring different aspects of daily life – family life, school/college, any treatment, limitations, participation in activities etc)
5. Which aspects of daily-living really matter to a family living with ESKD?
6. What are their experiences and beliefs around preparing for/receiving a kidney transplant?

[3] Conclusion:

5. *Summarise what has been discussed, mention the positive details, thank the young person / parent / carer*
6. "How did you find talking about living with chronic kidney disease?"
7. "Is there anything important to YOU that we have not talked about?"
8. Handle any concerns they have, signpost to any local services that could support their concerns.

For peer review only - http://bmjopen.bmj.com/site/about/guidelines.xhtml

An investigation of the modifiable psychosocial variables influencing access to and outcomes after kidney transplantation in children

Interview Topic Guide for Researcher – with NHS professional

Research Goals of the Interview:

7. Explore what the participant thinks matters most to their patients and families in terms of living a good quality life
8. Explore what the participant believes is delaying or enabling families to receive a kidney transplant from a mental health and social factors perspective

[1] Introduction, Overview:

- *Welcome, introduction of the research project and the researcher*
- *Establish a 'stop sign' for research participant to use if needing to pause the interview*

[2] Discussion of…:

Note for Interviewer: Subjects covered in this topic guide are to be explored openly – not leading. Appropriate prompts will be used – e.g. can you tell me more about that? Why do you think that is? How does that make you feel as a professional caring for these families?

7. From their experience of caring for families with ESKD, which aspects of daily-living really matter to these families?
8. What are their experiences of deciding whether a family is ready to prepare for kidney transplantation?
9. What are their experiences of families that do well and not so well after kidney transplantation?

[3] Conclusion:

9. *Summarise what has been discussed, mention the positive details, thank the NHS professional*
10. "How did you find the interview experience?"
11. "Is there anything important to YOU that we have not talked about?"
12. Handle any concerns they have, signpost to any local services that could support their concerns.

For peer review only - http://bmjopen.bmj.com/site/about/guidelines.xhtml

Supplementary Material 2

Demographic Background Survey

1. **What is your child's ethnic group?**
   a. White
   b. Black / Black-British
   c. Asian or Asian-British
   d. Mixed ethnicity
   e. Other ethnicity

2. **How many main caregivers (e.g. parents) are in your family?**
   a. One
   b. Two – living in same household
   c. Two – living in separate households
   d. Other _____________

3. **What do you identify yourself as in relation to your child?**
   a. Mother
   b. Father
   c. Aunt
   d. Uncle
   e. Grand mother
   f. Grand father
   g. Foster-carer
   h. Other _____________

4. **What does the other main caregiver identify themselves as in relation to your child? (if applicable)**
   a. Mother
   b. Father
   c. Aunt
   d. Uncle
   e. Grand mother
   f. Grand father
   g. Foster-carer
   h. Other _____________
   i. Not applicable

5. **What is the highest level of education you have completed?**
   a. Primary school
   b. Secondary school up to 16 years
   c. Higher or secondary or further education (e.g. A-levels, IB, BTEC)
   d. College or University
   e. Post-graduate degree

6. **What is the highest level of education, which the other main caregiver has completed? (If applicable)**
   a. Primary school
   b. Secondary school up to 16 years
   c. Higher or secondary or further education (e.g. A-levels, IB, BTEC)
   d. College or University
   e. Post-graduate degree
   f. Not applicable

7. **What is your current employment status?**
   a. Employed – full time
   b. Employed – Part time

For peer review only - http://bmjopen.bmj.com/site/about/guidelines.xhtml

c. Seeking employment
d. Home maker
e. Retired
f. Studying
g. Not seeking employment due to health reasons
h. Not seeking employment due to being full-time carer
i. Not seeking employment due to other reasons ________

8. What is the other main caregiver's current employment status? (If applicable)

a. Employed – full time
b. Employed – Part time
c. Seeking employment
d. Home maker
e. Retired
f. Studying
g. Not seeking employment due to health reasons
h. Not seeking employment due to being full-time carer
i. Not seeking employment due to other reasons __________
j. Not applicable

9. **Is there a recent family history of immigration into the United Kingdom?**

a. Yes, during my child or their sibling's lifetime (first generation)
b. Yes, during their parent's lifetime (second generation)
c. No recent history.

10. Have you had to move homes after your child was diagnosed with Chronic Kidney Disease?

a. Yes, home was inadequate for a child with Chronic Kidney Disease (e.g. unable to store dialysis equipment)
b. Yes, home was too far from child's hospital where they received care for their kidneys
c. Yes, but only temporarily (e.g. friend or relative's home or other temporary housing) because ____________
d. Yes, for other reasons _________________
e. Not applicable to our family

11. What is your family's total estimated income in the last year? (before tax, from all sources including salaries and benefits) Chronic Kidney Disease can have a knock-on effect on a family's circumstances, which is why we would like to understand this better by asking this information.

a. Up to £10,000
b. £10,001 to £20,000
c. £20,001 to £30,000
d. £30,001 to £50,000
e. £50,001 to £70,000
f. £70,001 to £150,000
g. Over £150,000
h. Prefer not to say

12. Next, we would like to ask you questions about your child's medications as this may have an effect on their quality of life.
Please select all of the kidney medicines your child is taking at the moment from this menu

- Aciclovir
- Azathioprine
- Calcium Acetate
- Calcium Carbonate
- Captopril
- Cefaclor
- Cefixim
- Ciclosporin (Sandimunn)
- Co-trimoxazole

For peer review only - http://bmjopen.bmj.com/site/about/guidelines.xhtml

- Darbepoetin
- Enalapril
- Epoetin
- Everolimus (Certican)
- Ganciclovir
- Growth Hormone
- Irbesartan
- Iron (Ferrous fumarate, Ferrous sulphate)
- Lactulose
- Losartan
- Movicol
- Mycophenolate Mofetil (MMF) (Cellcept, Myfenax)
- Nitrofurantoin
- Potassium Chloride
- Potassium Citrate
- Prednisolone
- Senna
- Sevelamer
- Sirolimus (Rapamune)
- Sodium Bicarbonate
- Sodium Chloride
- Tacrolimus (Advagraf, Prograf, Modigraf)
- Trimethoprim
- Valaciclovir
- Valganciclovir
- Vitamin D (Alfacalcidol)
- Vitamin D (Colecalciferol)
- Other(s)________ ________________

13. Please select how often your child takes these medicines (select all that apply):

a. Longer than every month
b. Every month
c. Every other week
d. Every week
e. Three times a week
f. Every other day
g. Once a day
h. Twice a day
i. Three times a day
j. Four times a day
k. More than four times a day
l. Other regime __________

14. Please select how these medicines are given to your child (select all that apply)

a. An injection directly into their vein (via cannula or other access to their veins)
b. Other type of injection (e.g. into skin or muscle)
c. Tablets (by mouth)
d. Tablets (by gastrostomy tube)
e. Liquids (by mouth)
f. Liquids (by gastrostomy tube)
g. Eye drops
h. Creams / Lotions
i. Inhaler
j. Other type of medicine ____________

For peer review only - http://bmjopen.bmj.com/site/about/guidelines.xhtml

15. **Some families may have had their journey towards a kidney transplant affected by the COVID-19 pandemic. Please select which situation applies to your child and family due to the COVID-19 pandemic in 2020-2021:**
    a. Our family was not in the middle of preparing for a kidney transplant during 2020-2021. Therefore COVID-19 did not impact us.
    b. Our family already started or were starting to prepare for a kidney transplant in 2020-2021. The COVID-19 pandemic did not change our preparation plans.
    c. Our family delayed preparations for a kidney transplant from a living donor due to the COVID-19 pandemic.
    d. Our family came off the waiting list for a kidney transplant from a deceased donor due to the COVID-19 pandemic
    e. Our family delayed investigations that were part of preparing for a kidney transplant overall because of the COVID-19 pandemic
    f. Our family's transplant journey was affected by the COVID-19 pandemic is other ways ___________________________

For peer review only
